# Bioactive Metabolites from the Mariana Trench Sediment-Derived Fungus *Penicillium* sp. SY2107

**DOI:** 10.3390/md18050258

**Published:** 2020-05-14

**Authors:** Sidra Kaleem, Le Qin, Wenwen Yi, Xiao-Yuan Lian, Zhizhen Zhang

**Affiliations:** 1 Ocean College, Zhoushan Campus, Zhejiang University, Zhoushan 316021, China; kaleemsidra85@yahoo.com (S.K.); qinle19951102@126.com (L.Q.); 18790658213@163.com (W.Y.); 2 College of Pharmaceutical Sciences, Zhejiang University, Hangzhou 310058, China

**Keywords:** hadal fungus, *Penicillium* sp. SY2107, andrastone C, antimicrobial activities

## Abstract

Mariana Trench sediments are enriched in microorganisms, however, the structures and bioactivities of their secondary metabolites are not very known. In this study, a fungus *Penicillium* sp. SY2107 was isolated from a sample of Mariana Trench sediment collected at a depth of 11000 m and an extract prepared from the culture of this fungus in rice medium showed antimicrobial activities. Chemical investigation on this active extract led to the isolation of 16 compounds, including one novel meroterpenoid, named andrastone C. Structure of the new compound was elucidated based on high-resolution electrospray ionization mass spectroscopy (HRESIMS) data, extensive nuclear magnetic resonance (NMR) spectroscopic analyses and a single crystal X-ray diffraction. The crystal structure of a known meroterpenoid andrastone B was also reported in this study. Both andrastones B and C exhibited antimicrobial activities against methicillin-resistant *Staphylococcus aureus* (MRSA), *Escherichia coli*, and *Candida albicans* with minimum inhibitory concentration (MIC) values in a range from 6 to 13 μg/mL.

## 1. Introduction

Marine natural products are important sources for the discovery of novel bioactive agents and drug leads [1,2,3,4,5]. However, the vast majority of these reported marine natural products are obtained from the shallow-water samples and only circa 2% are isolated from the deep-sea organisms [6,7,8]. This statistic contrasts significantly with that of the knowledge that 95% of the Earth’s seas are greater than 1000 m deep and the main reason for this disparity is the limitations in obtaining deep-sea organisms [7,8]. With the developments in technology to access deep-sea organisms, more and more deep-sea natural products have been reported [7,8,9].

The deep-sea organisms under extreme conditions have had to make significant biochemical and physiological adaptations for survival, which results in the modifications of both gene regulation and metabolic pathways to produce metabolites with unique structures and bioactivities that differ from those produced by the shallow-water organisms [7,8]. It was reported that about 75% of deep-sea natural products possess biological activity, about 40% were drug-like, and two/three were within Known Drug Space (KDS) [8]. For example, the marine obligate *Salinospora* actinomycetes are found in tropical and subtropical marine sediments at the depth of up to 1100 m [10,11]. The genus *Salinispora* has become a robust model for natural product research and the secondary metabolites reported to date from the *Salinospora* actinomycetes are predominantly new, including salinosporamide A, a second-generation proteasome inhibitor [12]. Salinosporamide A is currently termed as marizomib under investigation in malignant glioma and relapsed-refractory multiple myeloma [13,14].

**Figure 1:**
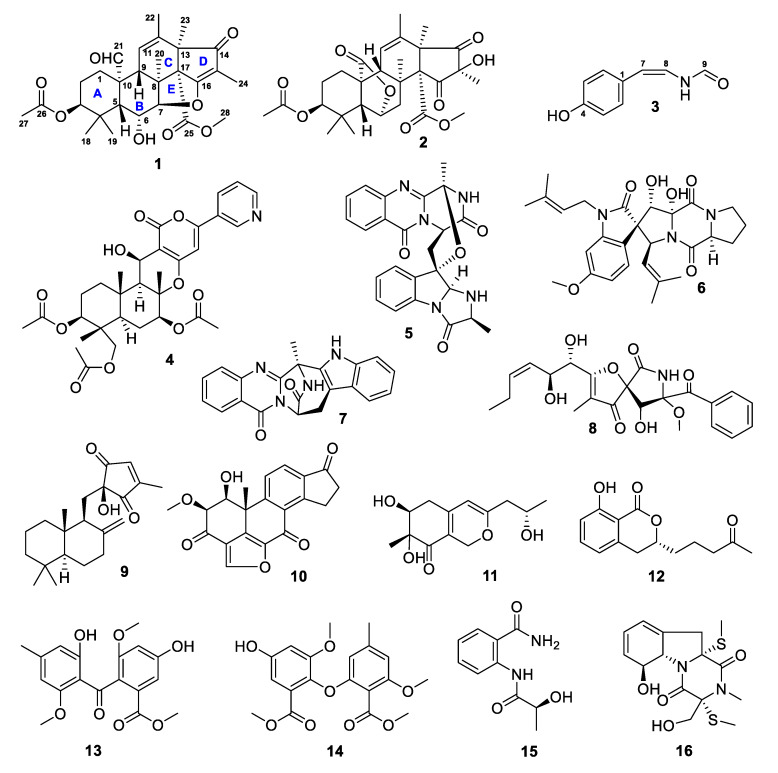
Structures of compounds **1**–**16** isolated from the culture of *Penicillium* sp. SY2107.

Mariana Trench sediments are enriched in microorganisms [15,16], however, the structures and bioactivities of their secondary metabolites are not very known and need to be explored. During the course of our ongoing project for the discovery of novel bioactive agents from marine microorganisms [17,18,19,20,21], a fungus strain SY2107 was isolated from a sediment sample collected from the Mariana Trench at depth of 11000 m. The extract prepared from the culture of this isolated hadal fungus in rice medium showed activities in inhibiting the growth of methicillin-resistant *Staphylococcus aureus* (MRSA), *Escherichia coli*, and *Candida albicans*. Chemical investigation on this active extract resulted in the isolation of 16 compounds (**1**–**16**, Figure 1), including one novel antimicrobial meroterpenoid, named andrastone C (**1**). Herein, we describe the isolation, structure elucidation, and bioactive evaluation of these isolated marine natural products.

## 2. Results and Discussion

The hadal fungus SY2107 (Figure S1, Supplementary Materials) was identified as *Penicillium* sp. SY2107 according to the result from its internal transcribed spacer (ITS) rDNA sequence (552 bp, Figure S2) analysis, which was 100% match to those of several *Penicillium* fungi (Table S1). An extract prepared from the culture of strain SY2107 in rice medium was separated by column chromatography, followed by high performance liquid chromatography (HPLC) purification, to afford compounds **1**–**16**.

**Figure 2:**
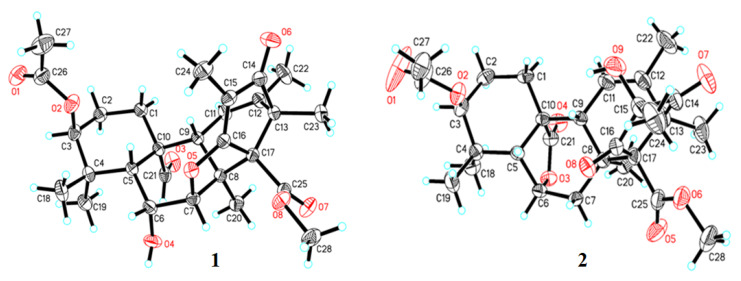
X-ray crystal structures of andrastones C (**1**) and B (**2**) (Cu Kα radiation).

Based on their high-resolution electrospray ionization mass spectroscopy (HRESIMS) data, extensive NMR spectroscopic analyses, and a single crystal X-ray diffraction as well as the comparison to the reported data, isolate **1** was elucidated as new meroterpenoid and **2**–**16** were identified as known compounds: andrastone B (16-*epi*-citreohybriddione A, **2**) [22,23], (*Z*)-*N*-(4-hydroxystyryl)formamide (**3**) [24,25], pyripyropene A (**4**) [26], fumiquinazoline C (**5**) [27], spirotryprostatin C (**6**) [28], fumiquinazoline J (**7**) [29], pseurotin A (**8**) [30], penicilliumin B (**9**) [31], (–)-viridin (**10**) [32], monascusone A (**11**) [33], aspergillumarin A (**12**) [34], 1,2-seco-trypacidin (**13**) [35], di-Me 2,3’-dimethylosoate (**14**) [36], 2*S*-(2-hydroxypropanamido) benzamide (**15**) [37], and bisdethiobis (methylthio)gliotoxin (**16**) [38]. The ^13^C and ^1^H NMR data of **2**–**16** were reported in Tables S2–S7. Andrastone B (**2**) is a meroterpenoid recently isolated from a deep-sea-derived fungus *Penicillium allii-sativi* [22,23] and its crystal structure (Figure 2) [Cu Kα radiation, Flack/Hooft parameter: −0.01(11)/0.08(10)] was reported in this study for the first time.

**Figure 3:**
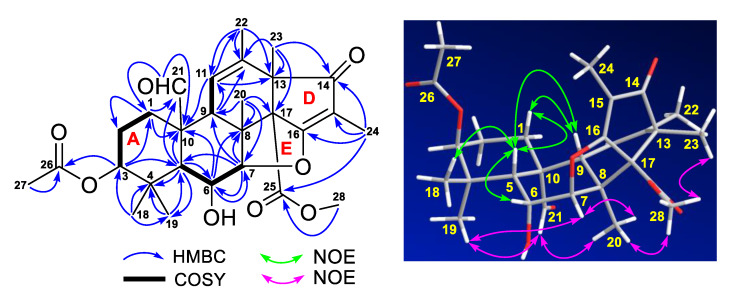
Correlation spectroscopy (COSY), key heteronuclear multiple bond correlation (HMBC), and nuclear Overhauser effect (NOE) correlations of andrastone C (**1**).

Compound **1** was obtained as monoclinic crystals and had a molecular formula C_28_H_36_O_8_ deduced from its HRESIMS ions at *m/z* 499.2340 [M – H]^–^ (calcd for C_28_H_35_O_8_, 499.2332) and 501.2482 [M + H]^+^ (calcd for C_28_H_37_O_8_, 501.2488). The infrared radiation (IR) spectrum showed characteristic bands for hydroxy (ν_max_ 3526 cm^−1^) and carbonyl (ν_max_ 1736, 1716, 1698, and 1660 cm^−1^) functional groups. The downfield ^13^C NMR spectrum showed eight signals for four carbonyls (*δ*_C_ 207.7, 201.1, 169.6, 168.3) and two pairs of double bonds (*δ*_C_ 185.3, 133.9, 120.5, 111.6) (Table 1). These NMR data accounted for six out of the 11 degrees of unsaturation required by the molecular formula and the remaining five suggested that the structure of **1** had five rings. In the correlation spectroscopy (COSY) spectrum, three spin systems of H_2_-2 (*δ*_H_ 1.47, m) with H_2_-1 (*δ*_H_ 2.31, dt, 13.5, 3.2 Hz; 0.79, td, 13.5, 4.5 Hz) and H-3 (*δ*_H_ 4.42, t, 3.1 Hz), H-6 (*δ*_H_ 4.52, t, 2.8 Hz) with H-5 (1.79, d, 2.8 Hz) and H-7 (*δ*_H_ 4.67, d, 2.8Hz), and H-9 (*δ*_H_ 2.05, br s) with H-11 (*δ*_H_ 5.60, br s) were observed (Figure 3). Heteronuclear multiple bond correlation (HMBC) spectrum showed the following correlations (Figure 3): H-1 (*δ*_H_ 0.79) with C-2 (*δ*_C_ 22.4), C-10 (*δ*_C_ 51.5), and C-21 (*δ*_C_ 207.7); H-3 with C-1 (*δ*_C_ 28.5), C-5 (*δ*_C_ 45.8), and C-26 (*δ*_C_ 169.6); H-5 with C-4 (*δ*_C_ 36.8), C-6 (*δ*_C_ 66.0), C-10, C-19 (*δ*_C_ 23.0), and C-21; H-6 with C-7 (*δ*_C_ 91.9), C-8 (*δ*_C_ 45.6), and C-10; H-7 with C-5, C-6, C-8, and C-9 (*δ*_C_ 50.1); H-9 with C-11 (*δ*_C_ 120.5) and C-12 (*δ*_C_ 133.9); H-11 with C-10, C-13 (*δ*_C_ 52.9), and C-22 (*δ*_C_ 20.2); H_3_-18 (*δ*_H_ 0.97, s) with C-3 (*δ*_C_ 77.4), C-4, C-5, and C-19; H_3_-19 (*δ*_H_ 1.09, s) with C-3, C-4, C-5, and C-18 (*δ*_C_ 25.3); H_3_-20 (*δ*_H_ 1.12, s) with C-7, C-8, C-9, and C-17 (*δ*_C_ 68.5); H_3_-21 (*δ*_H_ 10.55, s) with C-1, C-10, and C-20 (*δ*_C_ 15.5); H_3_-22 (*δ*_H_ 1.75, s) with C-11, C-12, and C-13; H_3_-23 (*δ*_H_ 1.13, s) with C-12, C-13, and C-17; and H_3_-27 (*δ*_H_ 1.93, s) with C-26. These COSY and HMBC correlations established the partial structure of rings A–C (a 6/6/6 tricyclic fusion) with an acetyl group at C-3, two oxymethines at C-6 and C-7, an aldehyde group at C-21, and five methyls at C-18, C-19, C-20, C-22, and C-23, respectively. In addition, HMBC correlations of H_3_-23 with C-12, C-13, C-14 (*δ*_C_ 201.1), and C-17, H_3_-24 (*δ*_H_ 1.57, s) with C-14, C-15 (*δ*_C_ 111.6), C-16 (*δ*_C_ 185.3), and C-25 (*δ*_C_ 168.3), and H_3_-28 (*δ*_H_ 3.69, s) with C-25 were also observed, indicating the C/D/E ring juncture with a keto at C-14, a methyl at C-24, and a methoxy at C-28. Although no HMBC correlation of H-7 with C-16 was observed, the downfield chemical shifts at *δ*_C_ 91.9 for C-7 and *δ*_C_ 185.3 for C-16 suggested a five-membered ether ring for E, which was confirmed by the crystal structure (Figure 2) of **1** obtained from a single crystal X-ray diffraction.

The relative configuration of **1** was assigned by nuclear Overhauser effect spectroscopy (NOESY) experiment. NOE correlations (Figure 3) of H-1β (*δ*_H_ 0.79) with H-5 and H-9 and H-5 with H-6, H-9, and H_3_-18 indicated a β-orientation for these protons, while NOE correlations of H-7 with H_3_-19 and H_3_-20, H_3_-21 with H_3_-19 and H_3_-20, and H_3_-28 with H_3_-20 and H_3_-23 were suggestive of a α-orientation for these protons. The relative configuration was confirmed and the absolute configuration of **1** was determined as 3*S*,5*R*,6*R*,7*R*,8*S,9R,10R,13R,17R* by a single crystal X-ray diffraction analysis [Cu Kα radiation, Flack/Hooft parameter: −0.02(8)/−0.00(6)] (Figure 2). Based on the foregoing evidences, compound **1** was elucidated as a new analogue of andrastone B (**2**), named andrastone C. Its ^13^C and ^1^H NMR data (Table 1) were assigned based on the heteronuclear multiple quantum correlation (HMQC), COSY, HMBC, and NOE correlations. To the best of our knowledge, andrastone C is the first example of this type of meroterpenoids with a five-membered ether ring (ring E).

Compounds **1**–**16** were tested for their antimicrobial activities against methicillin-resistant *Staphylococcus aureus* (MRSA), *Escherichia coli*, and *Candida albicans* by the micro-broth dilution method [39]. Vancomycin (an antibiotic against MRSA), gentamicin (an antibiotic against both Gram-positive and negative bacteria), and amphotericin B (an antifungal drug) were used as positive controls. The results (Table 2) showed that both andrastones C (**1**) and B (**2**) had antimicrobial activities with minimum inhibitory concentration (MIC) values of 8 and 9 μg/mL against MRSA, 8 and 12 μg/mL against *E. coli*, and 13 and 6 μg/mL against *C. albicans*, respectively. Known compounds **3**–**12** also showed antimicrobial activities against MRSA, *E. coli*, and *C. albicans* with MIC values in a range from 9 to 22 μg/mL, while compounds **13**–**16** only showed antibacterial activities against MRSA and *E. coli* with MIC values of 22–38 μg/mL.

Compounds **1**–**3** were also evaluated for their activities in inhibiting the proliferation of human glioma U251 and U87MG cells using the sulforhodamine B (SRB) assay [40]. Doxorubicin (DOX, a chemotherapeutic drug) was used as a positive control. (*Z*)-*N*-(4-hydroxystyryl)formamide (**3**) exhibited antiproliferative activity against U251 and U87MG cells with IC_50_ values of 17.0 ± 2.9 and 39.8 ± 1.6 μM, respectively. Both andrastones C (**1**) and B (**2**) showed no antiproliferative activity at a concentration of 50 μM.

## 3. Materials and Methods

### 3.1. General Experimental Procedures

Optical rotation was measured on an Autopol I polarimeter (Rudolph Research Analytical). Ultraviolet (UV) and IR spectra were recorded on a METASH UV-8000 spectrometer (Shanghai METASH Instruments Co. Ltd., Shanghai, China) and a Bruker TENSOR II high performance FT-IR spectrometer (Bruker, Karlsruhe, Germany), respectively. HRESIMS data were obtained from an Agilent 6230 Time of Flight Liquid Chromatography/Mass Spectrometry (TOF LC/MS) spectrometer. NMR spectra were acquired on a JEOL 600 spectrometer (Japan) using standard programs and acquisition parameters and chemical shifts were expressed in *δ* (ppm). X-ray diffraction analysis was performed on an Xcalibur Atlas Gemini Ultra diffractometer (Agilent Technologies) with Cu Kα radiation (λ = 1.54184 Å) at 100 K. Silica gel (100–200 mesh, Qingdao Haiyang Chemical Co., China), octadecyl-functionalized silica gel (ODS, Cosmosil 75C_18_-Prep, Nacalai Tesque Inc., Japan), and Sephadex LH-20 (GE Healthcare, Sweden) were used for open column chromatography. High performance liquid chromatography (HPLC) separation was carried out on an Agilent 1260 HPLC system with a diode array detector (DAD) using a Zorbax SB-C_18_ column (250 × 9.4 mm, 5 μm, Agilent Technologies, Palo Alto, USA) or a CXTH LC-3000 HPLC system (Beijing Chuangxin Tongheng Science & Technology Co. Ltd. China) using a CT-30 column (Fuji-C_18_, 280 × 30 mm, 10 μm). All solvents used for this study were ordered from the Shanghai Lingfeng Co., Ltd. (Shanghai, China). Methicillin-resistant *Staphylococcus aureus* (MRSA) ATCC 43300, *Escherichia coli* ATCC 25922, and *Candida albicans* ATCC 10231 were provided by Drs. Zhongjun Ma, Pinmei Wang, and Bin Wu, respectively. Human glioma U251 (XB-0439) and U87MG (JDS-2568) cells were purchased from the Cell Bank of the Chinese Academy of Sciences. Vancomycin (>98.0%), gentamicin (99.6%), and amphotericin B (99.0%) were obtained from the Meilune Biotechnology Co. Ltd. (Dalian, China), and doxorubicin (DOX, >98.0%) from Sigma-Aldrich. Sea salt was bought from the Zhejiang Province Salt Industry Group Company, Ltd. Artificial seawater (sea salt 35 g, water 1 L) was made in the laboratory. Different culture media were prepared in the laboratory, including B solid medium (soluble starch 20 g, KNO_3_ 1 g, MgSO_4_·7H_2_O 0.5 g, NaCl 0.5 g, K_2_HPO_4_ 0.5 g, FeSO_4_ 0.01 g, agar 15 g, water 1 L, pH 6–7), BY solid medium (soluble starch 20 g, KNO_3_ 1 g, MgSO_4_·7H_2_O 0.5 g, NaCl 0.5 g, K_2_HPO_4_ 0.5 g, FeSO_4_ 0.01 g, agar 15 g, sea salt 35 g, water 1 L, pH 6–7), PDA (potato dextrose agar) medium (potatoes 200 g, glucose 20 g, agar 20 g, boiled into 1 L of water for 15 min, pH 6–7), PDAY medium (potatoes 200 g, glucose 20 g, agar 20 g, sea salt 35 g, boiled into 1 L of water for 15 min, pH 6–7), E solid medium (yeast 1.0 g, tryptone 5.0 g, FeCl_3_·6H_2_O 0.17 g, KH_2_PO_4_ 0.12 g, agar 15 g, water 1 L, pH 6–7), EY solid medium (yeast 1.0 g, tryptone 5.0 g, FeCl_3_·6H_2_O 0.17 g, KH_2_PO_4_ 0.12 g, agar 15 g, sea salt 35 g, water 1 L, pH 6–7), ISP2 solid medium (yeast extract 4 g, malt extract 10 g, dextrose 4 g, peptone 5 g, agar 20 g, water 1 L, pH 6–7), ISP2Y solid medium (yeast extract 4 g, malt extract 10 g, dextrose 4 g, peptone 5 g, agar 20 g, sea salt 35 g, water 1 L, pH 6–7), ISP4 solid medium (soluble starch 10 g, K_2_HPO_4_ 1 g, MgSO_4_·7H_2_O 1 g, NaCl 1 g, (NH_4_)_2_SO_4_ 2 g, CaCO_3_ 2 g, FeSO_4_ 1 mg, MnCl_2_ 1 mg, ZnSO_4_ 1 mg, agar 20 g, water 1 L, pH 6–7), ISP4Y solid medium (soluble starch 10 g, K_2_HPO_4_ 1 g, MgSO_4_·7H_2_O 1 g, NaCl 1 g, (NH_4_)_2_SO_4_ 2 g, CaCO_3_ 2 g, FeSO_4_ 1 mg, MnCl_2_ 1 mg, ZnSO_4_ 1 mg, agar 20 g, sea salt 35 g, water 1 L, and pH 6–7).

### 3.2. Isolation and Identification of Strain SY2107

Strain SY2107 was isolated from a sediment sample, which was collected from the Mariana Trench at depth 11000 m on November, 2018. Briefly, the sediment was air dried at 28 °C for 7 days and the dried sample (1.0 g) was diluted with sterile water to make dilutions of 10^−2^, 10^−3^, and 10^−4^ g/mL. Each dilution (200 µL) was covered on the surface of ten different media of B, BY, D, DY, E, EY, ISP2, ISP2Y, ISP4, and ISP4Y in Petri dishes and then incubated at 28 °C for 14 days. The single pure colony of SY2107 was picked from the 10^−2^ g/mL suspension in ISP2Y solid medium and then transferred to another ISP2Y solid medium plate. After growth for another 7 days at 28 °C, the single colony (SY2107) that grew well was transferred onto an ISP2Y solid medium slant and stored at 4 °C for further study.

The strain SY2107 was identified by internal transcribed spacer (ITS) rDNA sequence analysis conducted by Legenomics (Hangzhou, China). The ITS rDNA sequence of strain SY2107 was compared to those in the GenBank using nucleotide BLAST (Basic Local Alignment Search Tool) and the rDNA sequence data of strain SY2107 has been deposited in GenBank with accession number MT355647. The strain *Penicillium* sp. SY 2107 was preserved at the Laboratory of Institute of Marine Biology and Pharmacology, Ocean College, Zhoushan Campus, Zhejiang University, China.

### 3.3. Scale Up Culture of Strain SY2107

Pure colony of strain SY2107 from the ISP2Y solid medium slant was inoculated into a 500 mL Erlenmeyer flask, which contained 250 mL ISP2Y liquid medium and then incubated for 3 days in a shaker (180 rpm, 28 °C) to produce seed broth. The seed broth (10 mL) was then transferred into rice medium (40 g rice and 60 mL artificial seawater) in 500 mL Erlenmeyer flask and then all flasks were incubated at 28 °C for 30 days in a static state. A total of 200 cultured flasks were prepared for this study.

### 3.4. Isolation of Compounds **1–16**

The culture of strain SY2107 in rice medium in each flask was extracted with EtOAc (250 mL) three times. The combined EtOAc extract was dried in vacuo to give an extract (70 g). This extract was fractionated on a column (160 × 10 cm) of silica gel (1200 g) eluting with a mixture (1000 mL) of cyclohexane and EtOAc in different ratios (10:1, 51, 2: 1, 1:1, and 1:2) to give five fractions fractions A–E.

Fraction A was separated by using an Agilent 1260 HPLC system with a Zorbax SB-C_18_ column (250 × 9.4 mm, 5 μm; UV detection: 230 nm; mobile phase: MeOH/H_2_O, 65/35; flow rate: 1.0 mL/min) to give **10** (5.0 mg, t_R_ 29.6 min) and **14** (6.7 mg, t_R_ 34.5 min).

Fraction B was first separated on a column (450 × 25 mm) of ODS (150 g) eluting with 60%, 70%, and 80% MeOH (each 1000 mL) to yield three subfractions B_1_–B_3_, respectively. Subfraction B_1_ was further separated on the CT-30 column (UV detection: 210 nm; mobile phase: MeOH/H_2_O, 59/41; flow rate: 10 mL/min) to give **16** (15.2 mg, t_R_ 36.4 min). Subfraction B_2_ was also separated on the same CT-30 column using the same flow rate and same UV detection wavelength and a different mobile phase of MeOH/H_2_O (80/20) to give **4** (10.1 mg; t_R_ 18.6 min) and **13** (22.0 mg; t_R_ 27.5 min). In the same way, Subfraction B_3_ was separated on the CT-30 column (UV detection: 210 nm; mobile phase: MeOH/H_2_O, 67/33; flow rate: 10 mL/min) to give parts B_3a_ and B_3b_. Part B_3a_ was further separated via the Zorbax SB-C_18_ column (UV detection: 230 nm; mobile phase: ACN/H_2_O, 45/55; flow rate: 1.0 mL/min) to give **9** (7.0 mg, t_R_ 38.5 min) and **2** (2.5 mg; t_R_ 42.4 min). By using the same Zorbax SB-C_18_ column at the same flow rate with different UV detection of 254 nm and mobile phase of ACN/H_2_O (42/58), compounds **1** (2.8 mg, t_R_ 43.8 min) and **3** (7 mg, t_R_ 35.2 min) were purified from part B_3b._

Similarly, fraction C was fractionated on a CT-30 column (280 × 30 mm, 10 μm; UV detection: 210 nm; mobile phase: MeOH/H_2_O, 50/50; flow rate: 10 mL/min) by a CXTH LC-3000 HPLC system to afford parts C_1_ and C_2_. Compounds **12** (6.7 mg, t_R_ 19.7 min) and **15** (12.1 mg, t_R_ 25.7 min) were obtained from parts C_1_ and C_2_, respectively, by HPLC purification using the Zorbax SB-C_18_ column with UV detection of 210 nm and mobile phase of 55% MeOH/H_2_O at flow rate of 1.0 mL/min.

Fraction D was first fractionated on a column (500 × 25 mm) of Sephadex LH-20 (120 g) eluting with 50% (300 mL), 60% (450 mL), and 70% MeOH (200 mL) to give three subfractions D_1_–D_3_, respectively. Subfraction D_1_ was further separated by the CT-30 column (UV detection: 210 nm; mobile phase: MeOH/H_2_O, 62/38; flow rate: 10.0 mL/min) to give **11** (15.0 mg, t_R_ 26.8 min), **5** (10.8 mg, t_R_ 36.4 min), and **7** (5.3 mg, t_R_ 32.8 min). By using the same Zorbax SB-C_18_ column with the same UV detection of 210 nm and the same flow rate of 1.0 mL/min, but different mobile phases, compound **6** (5.3 mg, t_R_ 28.0 min, MeOH/H_2_O, 70/30) was purified from subfraction D_2_ and **8** (4.2 mg, t_R_ 17.9 min, MeOH/H_2_O, 50/50) from subfraction D_3_.

Andrastone C (**1**): Colorless monoclinic crystal; molecular formula C_28_H_36_O_8_; m.p. 118–120 °C; [α]_D_^20^ + 60° (*c* 0.10, MeOH); UV (MeOH) λ_max_ (log ε) 256 (3.58) nm; IR (MeOH)ν_max_ 3526, 1736, 1716, 1698, 1660, 1030 cm^−1^; ^13^C NMR data (150 MHz, in dimethylsulfoxide-*d*_6_), Table 1, ^1^H NMR data (600 MHz, in dimethylsulfoxide-*d*_6_), Table 1; HRESIMS *m/z* 499.2340 [M − H]^−^ (calcd for C_28_H_35_O_8_, 499.2332), 501.2482 [M + H]^+^ (calcd for C_28_H_37_O_8_, 501.2488), and 523.2308 [M + Na]^+^ (calcd for C_28_H_36_NaO_8_, 523.2308).

Crystal data of andrastone C (**1**): C_28_H_36_O_8_ (*M* = 500.57 g/mol): monoclinic, space group P2_1_ (no. 4), *a* = 8.58899(11) Å, *b* = 8.86131(11) Å, *c* = 16.7937(2) Å, *β* = 96.8005(12)°, *V* = 1269.17(3) Å^3^, *Z* = 2, *T* = 293(2) K, μ(Cu Kα) = 0.784 mm^−1^, *Dcalc* = 1.310 g/cm^3^, 7640 reflections measured (5.3° ≤ 2Θ ≤ 147.088°), 4316 unique (*R*_int_ = 0.0172, R_sigma_ = 0.0240) which were used in all calculations. The final *R*_1_ was 0.0311 (I > 2σ(I)) and *wR*_2_ was 0.0866 (all data). The crystal data and structure refinement parameters of **1** were also reported in Table S8. Crystallographic data of andrastone C (**1**) has been deposited at the Cambridge Crystallographic Data Centre (CCDC Number: 1979103). Copies of the data can be obtained free of charge from Cambridge Crystallographic Data Centre, 12, Union Road, Cambridge CB2 1EZ, U.K. [fax (+44)1223-336-033; or e-mail: data_request@ccdc.cam.ac.uk).

Andrastone B (**2**): Colorless orthorhombic crystal; molecular formula C_28_H_36_O_9_; m.p. 130–134 °C [α]_D_^20^ −88° (*c* 0.02, MeOH); ^13^C NMR data (150 MHz, in MeOH-*d*_4_), Table S2, ^1^H NMR data (600 MHz, in MeOH-*d*_4_), Table S3; HRESIMS *m/z* 515.2286 [M − H]^−^ (calcd for C_28_H_35_O_9_, 515.2281) and 539.2253 [M + Na]^+^ (calcd for C_28_H_36_NaO_9_, 539.2257).

Crystal data of andrastone B (**2**): C_28_H_38_O_10_ (*M* = 534.58 g/mol): orthorhombic, space group P2_1_2_1_2_1_ (no. 19), *a* = 9.1880(3) Å, *b* = 13.2582(5) Å, *c* = 22.8448(6) Å, *V* = 2782.87(16) Å^3^, *Z* = 4, *T* = 293(2) K, μ(Cu Kα) = 0.802 mm^−1^, *Dcalc* = 1.276 g/cm^3^, 13131 reflections measured (7.71° ≤ 2Θ ≤ 147.056°), 5414 unique (*R*_int_ = 0.0263, R_sigma_ = 0.0313) which were used in all calculations. The final *R*_1_ was 0.0438 (I > 2σ(I)) and *wR*_2_ was 0.1180 (all data). The crystal data and structure refinement parameters of **3** were also reported in Table S15. Crystallographic data of andrastone B (**2**) has been deposited at the Cambridge Crystallographic Data Centre (CCDC Number: 1976943). Copies of the data can be obtained free of charge from Cambridge Crystallographic Data Centre, 12, Union Road, Cambridge CB2 1EZ, U.K. [fax (+44)1223-336-033; or e-mail: data_request@ccdc.cam.ac.uk).

### 3.5. Antimicrobial Active Assay

The antimicrobial activities of all isolated compounds against methicillin-resistant *Staphylococcus aureus* (MRSA), *Escherichia coli*, and *Candida albicans* were tested by the micro-broth dilution method as describe in the previous study [37] with little modification. Vancomycin, gentamicin, and amphotericin B were used as positive controls. Briefly, 96-well plates were used to make dilutions of the tested compounds. The first serial dilution was followed to get a broad range of concentration for each compound. Initial concentration was 200 µg/mL and then by serial dilution with 50% DMSO, other concentrations of 100, 50, 25, 12.5, 6.25, 3.125, and 1.5625 µg/mL were achieved. The final volume was 200 μL. After that 2 μL from 10^8^ cfu/mL of culture was added and the plates were incubated at 37 °C for 12 h overnight. Minimum inhibitory concentration (MIC) that inhibited the growth of microorganisms and minimum bactericidal concentration (MBC) that completely killed microorganisms were recorded. Finally, based on results obtained, specific concentrations of each compound were prepared to get more accurate values of MIC and MBC.

### 3.6. Antiproliferative Active Assay

The Sulforhodamine B (SRB) assay [38] was applied to evaluate the activity of the tested compounds in inhibiting the proliferation of human glioma U251 and U87MG cells. Doxorubicin (DOX) was used as a positive control. Human glioma U251 and U87MG cells were cultured in DMEM (Dulbecco′s Modified Eagle Medium, Gibco) and MEM (Minimum Essential Medium, Gibco) and with 10% FBS (Fetal Bovine Serum, PAA Laboratories Inc.), respectively. All cells were incubated in a 5% CO_2_ humidified incubator at 37 °C and the cultured cells after the third generation were used for the experiments.

## 4. Conclusions

One novel meroterpenoid, named andrastone C, and fifteen known compounds with diverse structural classes, were discovered and characterized from the culture of a Mariana Trench sediment-associated fungus *Penicillium* sp. SY2107 in rice medium. Andrastone C and most of the known compounds showed antimicrobial activities against MRSA, *E. coli*, and *C. albicans*. (*Z*)-*N*-(4-hydroxystyryl) formamide exhibited antiglioma activity. Data from this study enriched the chemical and bioactive diversities of the secondary metabolites from the Mariana Trench-sourced microorganisms.

## Figures and Tables

**Table 1:** ^13^C and ^1^H NMR data of andrastone C (**1**, in dimethylsulfoxide-*d*_6_).

No.	^13^C, Type	^1^H (*J* in Hz)	No.	^13^C, Type	^1^H (*J* in Hz)
1	28.5, CH_2_	*α*: 2.31 dt (13.5, 3.2); *β*: 0.79, td (13.5, 4.5)	15	111.6, C	–
2	22.4, CH_2_	1.47, m	16	185.3, C	–
3	77.4, CH	4.42, t (3.1)	17	68.5, C	–
4	36.8, C	–	18	25.3, CH_3_	0.97, s
5	45.8, CH	1.79, d (2.8)	19	23.0, CH_3_	1.09, s
6	66.0, CH	4.52, t (2.8)	20	15.5, CH_3_	1.12, s
7	91.9, CH	4.67, d (2.8)	21	207.7, CH	10.55, s
8	45.6, C	–	22	20.2, CH_3_	1.75, s
9	50.1, CH	2.05, br s	23	20.5, CH_3_	1.13, s
10	51.5, C	–	24	5.4, CH_3_	1.57, s
11	120.5, CH	5.60, br s	25	168.3, C	–
12	133.9, C	–	26	169.6, C	–
13	52.9, C	–	27	20.6, CH_3_	1.93, s
14	201.1, C	–	28	52.4, CH_3_	3.69, s

Note: No.: number, *J*: coupling constant, s: singlet, d: doublet, m: multiplet, br s: broad singlet.

**Table 2:** Antimicrobial activities of compounds **1**–**16** (μg/mL).

Compounds	MRSA		*E. coli*		*Candida albicans*	
	MIC	MBC	MIC	MBC	MIC	MBC
**1**	8	15	8	13	13	17
**2**	9	15	12	19	6	10
**3**	13	18	10	20	9	16
**4**	14	20	13	18	14	20
**5**	15	22	9	16	12	18
**6**	11	20	9	14	13	20
**7**	15	20	16	23	15	21
**8**	17	23	16	22	10	18
**9**	12	18	13	21	15	24
**10**	20	26	22	28	14	26
**11**	9	15	11	19	17	28
**12**	15	26	14	23	18	28
**13**	28	36	22	30	>50	>50
**14**	33	39	38	42	>50	>50
**15**	32	41	34	45	>50	>50
**16**	27	33	26	32	>50	>50
Gentamicin	3	7	0.5	3	NT	NT
Vancomycin	0.5	3	NT	NT	NT	NT
Amphotericin B	NT	NT	NT	NT	3	6

MIC: minimum inhibitory concentration; MBC: minimum bactericidal concentration; NT: No testing.

## Supplementary Materials

The following are available online at https://www.mdpi.com/1660-3397/18/5/258/s1, Colonies of *Penicillium* sp. SY2107, Figure S2: ITS rDNA sequence of *Penicillium* sp. SY2107, Table S1: Score statistics for sequence alignment of strain SY2107, Table S2–S7: ^13^C and ^1^H NMR data of compounds **2**–**16**, Figure S3–S22: NMR and HRESIMS spectra of andrastone C (**1**), Table S8–S14: Crystal data of X-ray diffraction of andrastone C (**1**), Table S15–S21: Crystal data of X-ray diffraction of andrastone B (**2**).
