# Supplementary material for: Bioactive Metabolites from the Mariana Trench Sediment-Derived Fungus Penicillium sp. SY2107
Source: Mar Drugs. 2020 May 14;18(5):258. doi: 10.3390/md18050258 (PMC7281598; doi:10.3390/md18050258)
Supplement: Supplementary file 1 [file marinedrugs-18-00258-s001.pdf]

SUPPLEMENTARY MATERIALS FOR

Bioactive Metabolites from the Mariana Trench Sediment-Derived Fungus *Penicillium* sp. SY2107

Sidra Kaleem [1], Le Qin [1], Wenwen Yi [1], Xiao-Yuan Lian [2,*] and Zhizhen Zhang [1,*]

[1] Ocean College, Zhoushan Campus, Zhejiang University, Zhoushan 316021, China; kaleemsidra85@yahoo.com (S.K.), qinle19951102@126.com (L.Q.), 18790658213@163.com (W.Y.)

[2] College of Pharmaceutical Sciences, Zhejiang University, Hangzhou 310058, China;

* Correspondence: xylian@zju.edu.cn (X.L.); zzhang88@zju.edu.cn (Z.Z.); Tel.: +86-13575476388 (X.L.); +86-13675859706 (Z.Z.)

CONTENT

Figure S_1. Colonies of *Penicillium* sp. SY2107 3
Figure S_2. ITS rDNA sequence of *Penicillium* sp. SY2107 3
Table S_1. Score statistics for sequence alignment of strain SY2107 4
Physicochemical data of known compounds **3–16**...4
Table S_2. ^{13}C NMR data of compounds **2–8**...7
Table S_3. ^{1}H NMR data of compounds **2–5**..8
Table S_4. ^{1}H NMR data of compounds **6–8**..9
Table S_5. ^{13}C NMR data of compounds **9–16**..10
Table S_6. ^{1}H NMR data of compounds **9–12**...11
Table S_7. ^{1}H NMR data of compounds **13–16**..12
Figures $S_{3\text{-}5}$. ^{1}H NMR spectra of andrastone C (**1**)..13
Figures $S_{6\text{-}9}$. ^{13}C NMR spectra of andrastone C (**1**) 14
Figures $S_{10\text{-}12}$. DEPT spectra of andrastone C (**1**) 16
Figures $S_{13\text{-}15}$. HMQC spectra of andrastone C (**1**) 18
Figure S_{16}. COSY spectrum of andrastone C (**1**)..19
Figures $S_{17\text{-}20}$. HMBC spectra of andrastone C (**1**) 20
Figure S_{21}. NOE spectrum of andrastone C (**1**)...22
Figure S_{22}. HRESIMS spectrum of andrastone C (**1**) 22
Table S_8. Crystal data and structure refinement parameters of andrastone C (**1**)...............23
Table S_9. Fractional atomic coordinates ($\times10^4$) and equivalent isotropic displacement parameters ($Å^2\times10^3$) for andrastone C (**1**). U_{eq} is defined as 1/3 of the trace of the orthogonalised U_{IJ}tensor..24
Table S_{10}. Anisotropic displacement parameters ($Å^2\times10^3$) for andrastone C (**1**). The anisotropic displacement factor exponent takes the form: -$2\pi^2[h^2a^{*2}U_{11}+2hka^*b^*U_{12}+\ldots]$..25
Table S_{11}. Bond lengths for andrastone C (**1**)...26
Table S_{12}. Bond angles for andrastone C (**1**)..27
Table S_{13}. Torsion angles for andrastone C (**1**)...28
Table S_{14}. Hydrogen atom coordinates ($Å\times10^4$) and isotropic displacement parameters ($Å^2\times10^3$) for andrastone C (**1**)..30
Table S_{15}. Crystal data and structure refinement parameters of andrastone B (**2**)..............31
Table S_{16}. Fractional atomic coordinates ($\times10^4$) and equivalent isotropic displacement parameters ($Å^2\times10^3$) for andrastone B (**2**). U_{eq} is defined as 1/3 of the trace of the orthogonalised U_{IJ}tensor..32
Table S_{17}. Anisotropic displacement parameters ($Å^2\times10^3$) for andrastone B (**2**). The anisotropic displacement factor exponent takes the form: -$2\pi^2[h^2a^{*2}U_{11}+2hka^*b^*U_{12}+\ldots]$...........................33
Table S_{18}. Bond lengths for andrastone B (**2**)...34
Table S_{19}. Bond Angles for andrastone B (**2**)..35
Table S_{20}. Torsion angles for andrastone B (**2**)...36
Table S_{21}. Hydrogen atom coordinates ($Å\times10^4$) and isotropic displacement parameters ($Å^2\times10^3$) for andrastone B (**2**)..38

Figure S_1. Colonies of *Penicillium* sp. SY2107 cultured in ISP2Y solid medium at 28 °C for 7 days

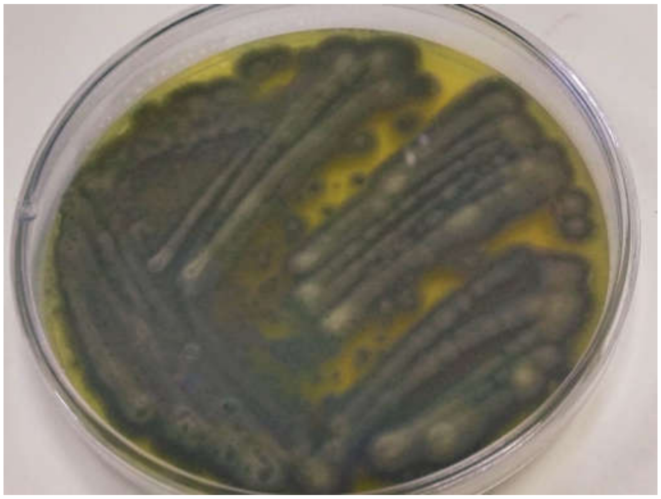

Figure S_2. ITS rDNA sequence of *Penicillium* sp. SY2107

GAAGGATCATTACCGAGTGAGGGCCCTCTGGGTCCAACCTCCCACCCGTGTTTA
TTTTACCTTGTTGCTTCGGCGGGCCCGCCTTAACTGGCCGCCGGGGGGCTTACG
CCCCCGGGCCCGCGCCCGCCGAAGACACCCTCGAACTCTGTCTGAAGATTGTA
GTCTGAGTGAAAATATAAATTATTTAAAACTTTCAACAACGGATCTCTTGGTTC
CGGCATCGATGAAGAACGCAGCGAAATGCGATACGTAATGTGAATTGCAAATT
CAGTGAATCATCGAGTCTTTGAACGCACATTGCGCCCCCTGGTATTCCGGGGGG
CATGCCTGTCCGAGCGTCATTGCTGCCCTCAAGCACGGCTTGTGTGTTGGGCCC
CGTCCTCCGATCCCGGGGGACGGGCCCGAAAGGCAGCGGCGGCACCGCGTCCG
GTCCTCGAGCGTATGGGGCTTTGTCACCCGCTCTGTAGGCCCGGCCGGCGCTTG
CCGATCAACCCGAATTTTTATCCAGGTTGACCTCGGATCAGGTAGGGATACCCG
CTGAACTTAAGCATA (552 bp)

Table S1. Score statistics for sequence alignment of strain SY2107

Accession	Description	Max score	Total score	Query coverage	Evalue	Ident
MN413181.1	*Penicillium* sp. strain Pb-G small subunit ribosomal RNA gene, 5.8S ribosomal RNA gene, and internal transcribed spacer 2	1020	1020	100%	0.0	100%
MK267450.1	*Penicillium chrysogenum* isolate E20401_ITS small subunit ribosomal RNA gene, 5.8S ribosomal RNA gene, and internal transcribed spacer 2	1020	1020	100%	0.0	100%
MK178856.1	*Penicillium chrysogenum* isolate Seq_MEL-07 small subunit ribosomal RNA gene, 5.8S ribosomal RNA gene,	1020	1020	100%	0.0	100%
MH865997.1	*Penicillium chrysogenum* strain CBS 132216, 5.8S ribosomal RNA gene	1020	1020	100%	0.0	100%
MF077262.1	*Penicillium chrysogenum* strain F-98 small subunit ribosomal RNA gene, internal transcribed spacer 1, 5.8S ribosomal RNA gene, and internal transcribed spacer 2	1020	1020	100%	0.0	100%
MF077261.1	*Penicillium chrysogenum* strain F-94 small subunit ribosomal RNA gene, partial sequence; internal transcribed spacer 1, 5.8S ribosomal RNA gene, and internal transcribed spacer 2	1020	1020	100%	0.0	100%

Physicochemical data of known compounds 3–16

(*Z*)-*N*-(4-hydroxystyryl)formamide (**3**): Colorless amorphous powder; molecular formula $C_9H_9NO_2$; UV (MeOH) λ_{max} (log ε) 278 (3.10), 217 (2.81) nm; IR (MeOH) ν_{max} 3253, 2920, 2850, 1653, 1604, 1484, 1394, 1231, 1204, 1180, 841 cm^{-1}; ^{13}C NMR data (150 MHz, in DMSO-d_6), Table S2, ^{1}H NMR data (600 MHz, in DMSO-d_6), Table S3; HRESIMS *m/z* 164.0711 [M+H]$^+$ (calcd for $C_9H_{10}NO_2$, 164.0712) and 186.0527 [M+Na]$^+$ (calcd for $C_9H_9NNaO_2$, 186.0531).

Pyripyropene A (**4**): White solid; molecular formula $C_{31}H_{37}NO_{10}$; $[\alpha]_D^{20}$ +42° (*c* 0.10, MeOH); ^{13}C NMR (data 150 MHz, in MeOH-d_4), Table S2, ^{1}H NMR data (600 MHz, in MeOH-d_4), Table S3.

Fumiquinazoline C (**5**): Light-yellow amorphous powder; molecular formula $C_{24}H_{21}N_5O_4$; $[\alpha]_D^{20}$ –60.8° (*c* 0.10, MeOH); ^{13}C NMR data (150 MHz, in DMSO-d_6), Table S2, ^{1}H NMR data (600 MHz, in DMSO-d_6), Table S3.

Spirotryprostatin C (**6**): Pale-yellow amorphous powder; molecular formula $C_{27}H_{33}N_3O_6$ $[\alpha]_D^{20}$ –52° (*c* 0.20, MeOH); ^{13}C NMR data (150 MHz, in CHCl$_3$-*d*), Table S2, ^{1}H NMR data (600 MHz, in CHCl$_3$-*d*), Table S4.

Fumiquinazoline J (**7**): White amorphous powder; molecular formula $C_{21}H_{16}N_4O_2$;

$[\alpha]_D^{20}$ –68° (*c* 0.10, MeOH), MeOH); ^{13}C NMR data (150 MHz, in DMSO-d_6), Table S2, ^{1}H NMR data (600 MHz, in DMSO-d_6), Table S4.

Pseurotin A (**8**): White amorphous powder; molecular formula $C_{22}H_{25}NO_8$; $[\alpha]_D^{20}$ –45° (*c* 0.20, MeOH); ^{13}C NMR data (150 MHz, in DMSO-d_6), Table S2, ^{1}H NMR data (600 MHz, in DMSO-d_6), Table S4.

Penicilliumin B (**9**): White crystal; molecular formula $C_{21}H_{30}O_3$; $[\alpha]_D^{20}$ +32° (*c* 0.20, MeOH); ^{13}C NMR data (150 MHz, in $CHCl_3$-*d*), Table S5, ^{1}H NMR data (600 MHz, in $CHCl_3$-*d*), Table S6.

(–)-Viridin (**10**): White crystals; molecular formula $C_{20}H_{16}O_6$; $[\alpha]_D^{20}$ –34° (*c* 0.20, MeOH); ^{13}C NMR data (150 MHz, in DMSO-d_6), Table S5, ^{1}H NMR data (600 MHz, in DMSO-d_6), Table S6.

Monascusone A (**11**): Yellow oil; molecular formula $C_{13}H_{18}O_5$; $[\alpha]_D^{20}$ +48° (*c* 0.20, MeOH); ^{13}C NMR data (150 MHz, in MeOH-d_4), Table S5, ^{1}H NMR data (600 MHz, in MeOH-d_4), Table S6.

Aspergillumarin A (**12**): Colorless oil; molecular formula $C_{14}H_{16}O_4$; $[\alpha]_D^{20}$ –39° (*c* 0.10, MeOH); ^{13}C NMR data (150 MHz, in $CHCl_3$-*d*), Table S5, ^{1}H NMR data (600 MHz, in $CHCl_3$-*d*), Table S6.

1,2-Seco-trypacidin (**13**): White powder; molecular formula $C_{18}H_{18}O_7$; ^{13}C NMR data (150 MHz, in DMSO-d_6), Table S5, ^{1}H NMR data (600 MHz, in DMSO-d_6), Table S7.

Di-Me 2,3'-dimethylosoate (**14**): White needle; molecular formula $C_{19}H_{20}O_8$; ^{13}C NMR data (150 MHz, in MeOH-d_4), Table S5, ^{1}H NMR data (600 MHz, in MeOH-d_4), Table S7.

(*S*)-2-(2-Hydroxypropanamido)benzamide (**15**): Light-yellow powder; molecular formula $C_{10}H_{12}N_2O_3$; $[\alpha]_D^{20}$ –34° (*c* 0.15, MeOH); ^{13}C NMR data (150 MHz, in DMSO-d_6), Table S5, ^{1}H NMR data (600 MHz, in DMSO-d_6), Table S7.

Bisdethiobis(methylthio)gliotoxin (**16**): Yellow oil; molecular formula $C_{15}H_{20}N_2O_4S_2$; $[\alpha]_D^{20}$ –21° (*c* 0.15, MeOH); ^{13}C NMR data (150 MHz, in $CHCl_3$-*d*), Table S5, ^{1}H NMR data (600 MHz, in $CHCl_3$-*d*), Table S7.

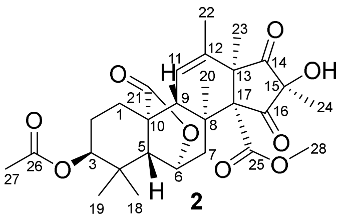

2

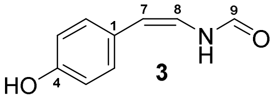

3

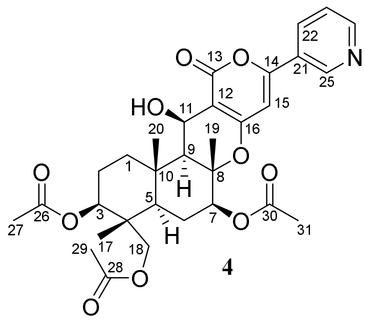

4

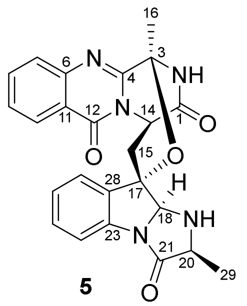

5

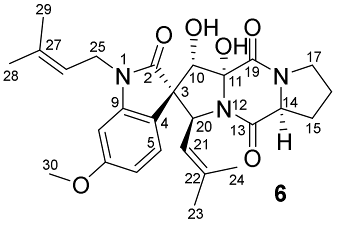

6

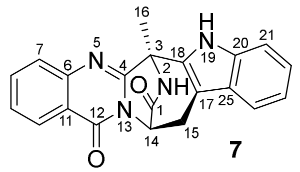

7

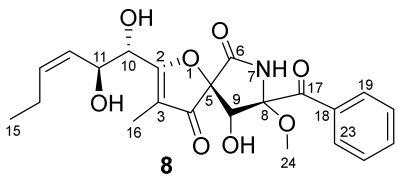

8

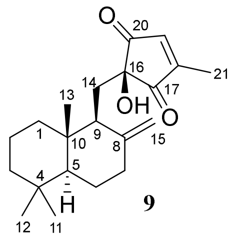

9

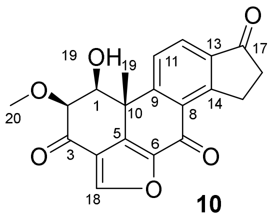

10

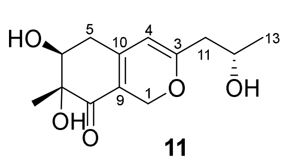

11

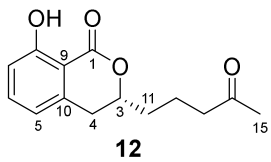

12

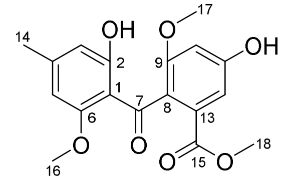

13

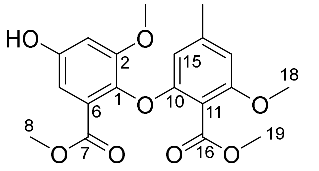

14

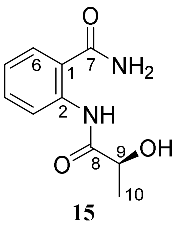

15

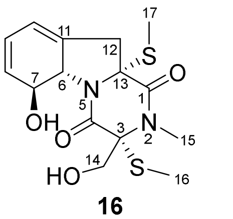

16

Table S2. ^{13}C NMR data of compounds **2**–**8** (150 MHz)

No.	**2**[a]	**3**[a]	**4**[a]	**5**[b]	**6**[c]	**7**[b]	**8**[b]
1	21.9, CH_2	127.9, C	37.2, CH_2	172.3, C	–	169.1, C	–
2	23.2, CH_2	130.8, CH	24.0, CH_2	–	178.9, C	–	166.6, C
3	77.5, CH	116.7, CH	75.4, CH	83.8, C	60.9, C	54.6, C	111.6, C
4	35.7, C	157.9, C	41.8, C	150.3, C	117.5, C	154.3, C	196.7, C
5	56.8, CH	116.7, CH	46.8, CH	–	126.9, CH	–	92.5, C
6	79.6, CH	130.8, CH	26.2, CH_2	146.3, C	106.6, CH	146.6, C	187.0, C
7	36.5, CH_2	114.0, CH	79.9, CH	128.0, CH	160.6, C	127.4, CH	–
8	41.0, C	118.7, CH	84.6, C	134.5, CH	97.2, CH	134.8, CH	91.2, C
9	53.4, CH	161.9, CH	55.6, CH	128.1, CH	144.6, C	127.4, CH	75.0, CH
10	45.4, C		39.3, C	126.1, CH	75.4, CH	126.4, CH	72.0, CH
11	125.9, CH		60.4, CH	121.1, C	87.2, C	119.4, C	68.2, CH
12	135.3, CH		104.7, C	159.3, C	–	159.3, C	129.1, CH
13	62.0, C		165.2, C	–	169.0, C	–	133.9, CH
14	209.4, C		158.4, C	51.1, CH	60.7, CH	54.1, CH	20.7, CH_2
15	73.1, C		101.1, CH	31.2, CH_2	27.7, CH_2	25.6, CH_2	14.2, CH_3
16	213.9, C		164.3, C	23.9, CH_3	23.3, CH_2	18.3, CH_3	5.7, CH_3
17	74.7, C		13.6, CH_3	87.0, C	45.0, CH_2	105.5, C	196.5, C
18	23.0, CH_3		66.2, CH_2	85.6, CH	–	134.0, C	133.8, C
19	26.8, CH_3		16.9, CH_3	–	165.1, C	–	130.3, CH
20	21.5, CH_3		18.1, CH_3	58.1, CH	57.6, CH	134.8, C	128.4, CH
21	181.3, C		129.3, C	170.0, C	121.8, CH	111.7, CH	133.5, CH
22	18.9, CH_3		135.1, CH	–	138.7, C	122.3, CH	128.4, CH
23	19.6, CH_3		125.6, CH	136.2, C	17.9, CH_3	120.2, CH	130.3, CH
24	20.1, CH_3		152.1, CH	114.5, CH	25.3, CH_3	118.1, CH	51.7, CH_3
25	169.4, C		147.6, CH	129.9, CH	38.6, CH_2	127.3, C	
26	172.4, C		172.1, C	125.5, CH	117.4, CH		
27	21.0, CH_3		21.2, CH_3	125.1, CH	137.7, C		
28	52.5, CH_3		172.7, C	138.3, C	18.2, CH_3		
29			20.8, CH_3	17.5, CH_3	25.6, CH_3		
30			172.6, C		55.6, CH_3		
31			21.2, CH_3				

[a,,b,c] The data were recorded in MeOH-d_4, DMSO-d_6, and $CHCl_3$-d, respectively.

Table S3. ^{1}H NMR data of compounds **2**–**5** (600 MHz, J in Hz)

No.	**2**[a]	**3**[a]	**4**[a]	**5**[b]
1	α: 2.10, dd (13.5, 3.6); β: 1.42, dt (13.5, 5.7)	–	a: 2.15, m; b: 1.27, m	–
2	a: 1.74, m; b: 1.66, m	7.18, d (8.5)	a: 1.89, m; b: 1.84, m	9.97, s
3	4.62, dd (3.7, 1.9)	6.79, d (8.5)	4.80, dd (11.9, 5.1)	–
5	1.95, br s	6.79, d (8.5)	1.61, m	–
6	4.91, d (4.0)	7.18, d (8.5)	a: 1.82, m; b: 1.66, m	–
7	α: 2.64, dd (14.5, 4.7); β: 1.42, d (14.5)	5.74, d (9.9)	4.98, m	7.78, d (8.0)
8	–	6.73, d (9.9)	–	7.87, td (8.0, 1.6)
9	2.73, t (2.3)	8.09, s	1.61, m	7.62, td (8.0, 1.6)
10	–		–	8.19, d (8.0)
11	5.79, br s		4.97, m	–
14	–		–	5.36, d (7.0)
15	–		6.80, s	a: 2.91 dd (15.3, 7.0); b: 2.04, d (15.3)
16	–		–	1.89, s
17	–		0.92, s	–
18	0.90, s		a: 3.79, d (11.9); b: 3.74, d (11.9)	5.08, d (9.3)
19	1.00, s		1.75, s	2.32, t (9.3)
20	1.40, s		1.49, s	3.50, m
21	–		–	–
22	1.72, d (2.3)		8.27, dd (8.2, 2.0)	–
23	1.35, s		7.53, dd (8.2, 5.1)	–
24	1.32, s		8.62, d (5.1)	7.33, d (7.6)
25	–		9.02, s	7.34, t (7.6)
26	–		–	7.20, t (7.6)
27	2.05, s		2.03, s	7.30, d (7.6)
28	3.62, s		–	–
29	–		2.07, s	0.87, d (7.2)
31			2.13	

[a,,b,c] The data were recorded in MeOH-d_4 and DMSO-d_6, respectively.

Table S4. ^{1}H NMR data of compounds **6–8** (600 MHz, *J* in Hz)

No.	**6**[a]	**7**[b]	**8**[b]
2	–	9.57, s	–
5	6.98, d (9.0)	–	–
6	6.56, dd (9.0, 2.7)	–	–
7	–	7.64, d (7.9)	9.96, s
8	6.42, d (2.7)	7.80, t (7.9)	–
9	–	7.52, t (7.9)	4.40, d (8.8)
10	4.82, s	8.14, d (7.9)	4.34, t (5.7)
11	–	–	4.45, m
12	–	–	5.40, t (7.7)
13	–	–	5.37, m
14	4.61, t (8.8)	5.70, dd (4.6, 3.0)	a: 2.02, m; b: 1.96, m
15	a: 2.38, m; b: 2.08, m	a: 3.41 dd (17.5, 3.0); b: 3.21, dd (17.5, 4.6)	0.87, t (7.7)
16	a: 2.05, m; b: 1.97, m	2.12, s	1.64, s
17	3.59, m	–	–
19	–	11.21, s	8.25, d (8.1)
20	4.85, d (9.0)	–	7.52, t (8.1)
21	4.90, d (9.0)	7.36, d (8.0)	7.66, t (8.1)
22	–	7.10, t (8.0)	7.52, t (8.1)
23	1.07, s	6.98, t (8.0)	8.25, d (8.1)
24	1.61, s	7.40, d (8.0)	3.24, s
25	a: 4.39, dd (15.6, 7.0); b: 4.22, dd (15.6, 7.0)	–	–
26	5.12, t (7.0)	–	–
28	1.83, s	–	–
29	1.73, s	–	–
30	3.82, s	–	–
OH-9	–	–	6.24, d (8.8)
OH-10	–	–	5.75, d (5.7)
OH-11	–	–	4.88, d (4.8)

[a,b] The data were recorded in $CHCl_3$-*d* and DMSO-d_6, respectively.

Table S_5. ^{13}C NMR data of compounds **9**–**16** (150 MHz)

No.	**9**[a]	**10**[b]	**11**[c]	**12**[a]	**13**[b]	**14**[c]	**15**[b]	**16**[a]
1	38.6, CH_2	72.3, CH	65.3, CH_2	170.0, C	110.1, C	136.1, C	121.5, C	165.8, C
2	19.3, CH_2	85.6, CH	–	–	163.3, C	157.3, C	139.3, C	–
3	41.9, CH_2	187.8, C	166.9, C	79.6, CH	103.5, CH	106.3, CH	123.0, CH	71.8, C
4	33.7, C	121.6, C	104.4, CH	33.0, CH_2	147.8, C	155.4, C	132.3, CH	166.7, C
5	55.5, CH	144.4, C	35.6, CH_2	118.2, CH	110.1, CH	109.2, CH	120.5, CH	–
6	24.4, CH_2	145.9, C	73.7, CH	136.4, CH	160.8, C	127.7. C	129.1, CH	69.6, CH
7	38.0, CH_2	172.9, C	78.3, C	116.4, CH	199.4, C	168.0, C	170.9, C	74.4, CH
8	149.7, C	129.2, C	199.2, C	162.4, C	125.8, C	52.8, CH_3	174.7, C	130.0, CH
9	51.2, CH	157.2, C	114.4, C	108.6, C	156.6, C	56.8, CH_3	68.5, CH	123.2, CH
10	40.0, C	41.9, C	151.4, C	139.5, C	103.2, CH	158.2, C	21.5, CH_3	120.1, CH
11	33.5, CH_3	128.1, CH	44.8, CH_2	34.2, CH_2	158.2, C	111.5, C	–	131.5, C
12	21.6, CH_3	126.5, CH	66.6, CH	19.2, CH_2	107.2, CH	159.0, C	–	39.0, CH_2
13	14.7, CH_3	136.4, C	18.3, CH_3	43.1, CH_2	127.9, C	106.0, CH	–	71.5, C
14	30.0, CH_2	157.2, C	23.6, CH_3	208.5, C	21.9, CH_3	143.0, C	–	63.6, CH_2
15	108.4, CH_2	28.2, CH_2	–	30.2, CH_3	165.8, C	107.8, CH	–	28.6, CH_3
16	75.5, C	35.9, CH_2	–	–	52.1, CH_3	169.1, C	–	13.7, CH_3
17	203.2, C	205.7, C	–	–	55.9, CH_3	22.2, CH_3	–	15.1, CH_3
18	160.4, C	29.8, CH_3	–	–	55.9, CH_3	56.6, CH_3	–	–
19	142.9, CH	151.5, CH	–	–	–	52.8, CH_3	–	–
20	201.6, C	60.5, CH_3	–	–	–	–	–	–
21	12.0, CH_3	–	–	–	–	–	–	–
22	–	–	–	–	–	–	–	–

[a,,b,c] The data were recorded in $CHCl_3$-*d*, DMSO-d_6, and MeOH-d_4, respectively.

Table S_6. ^{1}H NMR data of compounds **9**–**12** (600 MHz, *J* in Hz)

No.	**9**[a]	**10**[b]	**11**[c]	**12**[a]
1	a: 1.58, br d (12.4); b: 0.92, dt (12.4, 4.2)	4.38, d (4.5)	a: 4.86, d (12.8); b: 4.78, d (12.8)	–
2	a: 1.50, m; b: 1.46, m	3.76, d (4.5)	–	–
3	a: 1.34, br d (13.2); b: 1.13, dt (13.2, 4.2)	–	–	4.56, m
4	–	–	5.38, s	a: 2.94, dd (16.2, 10.3); b: 2.90, dd (16.2, 4.5)
5	1.10, dd (12.8, 2.9)	–	a: 2.63, dd (17.9, 5.4); b: 2.41, d (17.9, 10.1)	6.69, d (7.9)
6	a: 1.72, m; b: 1.66, m	–	3.90, dd (10.1, 5.4)	7.40, t (7.9)
7	a: 2.31, td (12.6, 2.9); b: 1.94, m	–	–	6.88, d (7.9)
8	–	–	–	–
9	1.86, d (14.2)	–	–	–
10	–	–	–	a: 1.86, m; b: 1.79, m
11	0.84, s	8.68, d (8.1)	a: 2.34, dd (14.2, 7.3); b: 2.28, d (14.2, 5.3)	a: 1.86, m; b: 1.79, m
12	0.75, s	7.92, d (8.1)	3.98, m	a: 1.83, m; b: 1.75, m
13	0.59, s	–	1.19, d (6.0)	2.53, t (7.1)
14	a: 2.01, dd (14.2, 10.6); b: 1.91, d (14.2)	–	1.22, s	–
15	a: 4.85, s; b: 4.54, s	a: 3.63, m; b: 3.52, m	–	2.17, s
16	–	2.66, m	–	–
18	–	1.64, s	–	–
19	6.93, s	8.97, s	–	–
20	–	3.58, s	–	–
21	2.09, s	–	–	–
OH-8	–	–	–	10.99, s

[a,,b,c] The data were recorded in $CHCl_3$-*d*, DMSO-d_6, and MeOH-d_4, respectively.

Table S7. ^{1}H NMR data of compounds **13–16** (600 MHz, *J* in Hz) **1 2**

No.	**13**^{b}	**14**^{c}	**15**^{b}	**16**^{a}
3	6.69, d (1.9)	6.72, d (2.8)	7.74, d (8.2)	–
4	–	–	7.46, t (8.2)	–
5	6.90, d (1.9)	6.80, d (2.8)	7.10, t (8.2)	–
6	–	–	8.57, d (8.2)	4.92, d (14.2)
7	–	–	–	4.89, d (14.2)
8	–	3.72, s	–	5.73, d (9.8)
9	–	3.68, s	4.10, m	5.87, dd (9.8, 4.7)
10	6.26, s	–	1.30, d (6.5)	5.93, d (4.7)
12	6.38, s	–	–	a: 3.06, d (16.0); b: 2.92, d (16.0)
13	–	6.48, s	–	–
14	2.26, s	–	–	a: 4.37, d (12.0); b: 3.90, d (12.0)
15	–	5.86, s	–	3.14, s
16	3.33, s	–	–	2.26, s
17	3.63, s	2.19, s	–	2.24, s
18	3.62, s	3.81, s	–	–
19	–	3.85, s	–	–
OH-2	12.97, s	–	–	–
OH-9	–	–	5.98, d (3.5)	–
NH-2	–	–	12.0, s	–
NH_2-7	–	–	a: 8.17, s; b: 7.70, s	–

^{a,,b,c} The data were recorded in $CHCl_3$-*d*, DMSO-*d*$_6$, and MeOH-*d*$_4$, respectively.

Figure S_3. ^1H NMR spectrum of andrastone C (**1**)

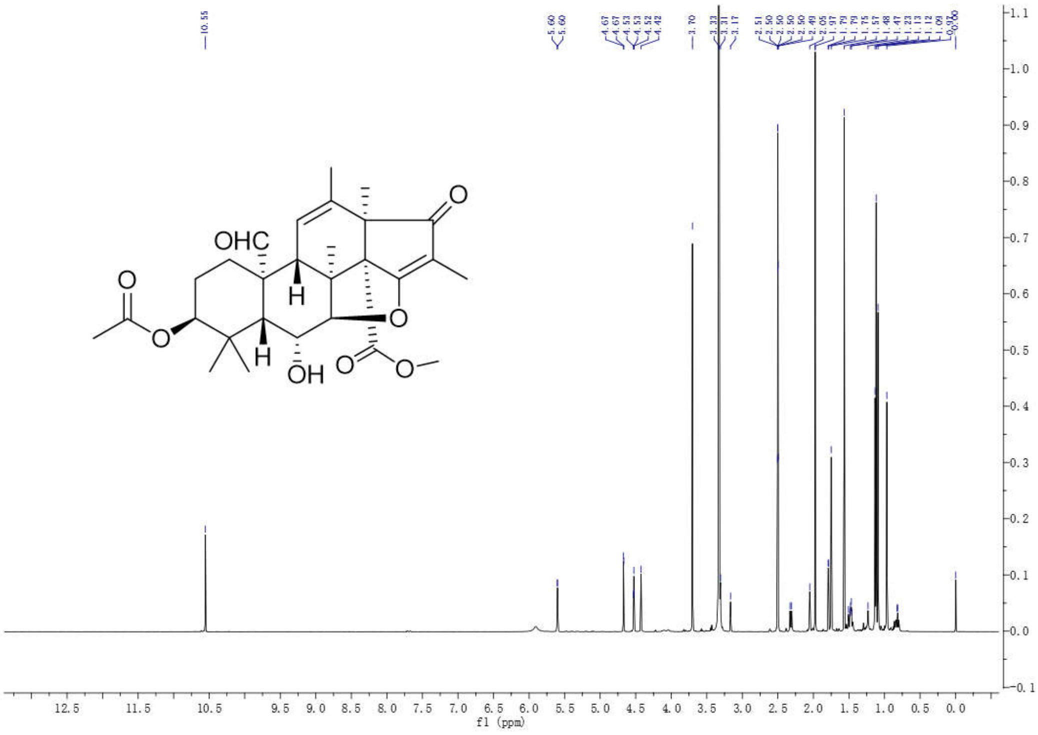

Figure S_4. ^1H NMR spectrum of andrastone C (**1**)

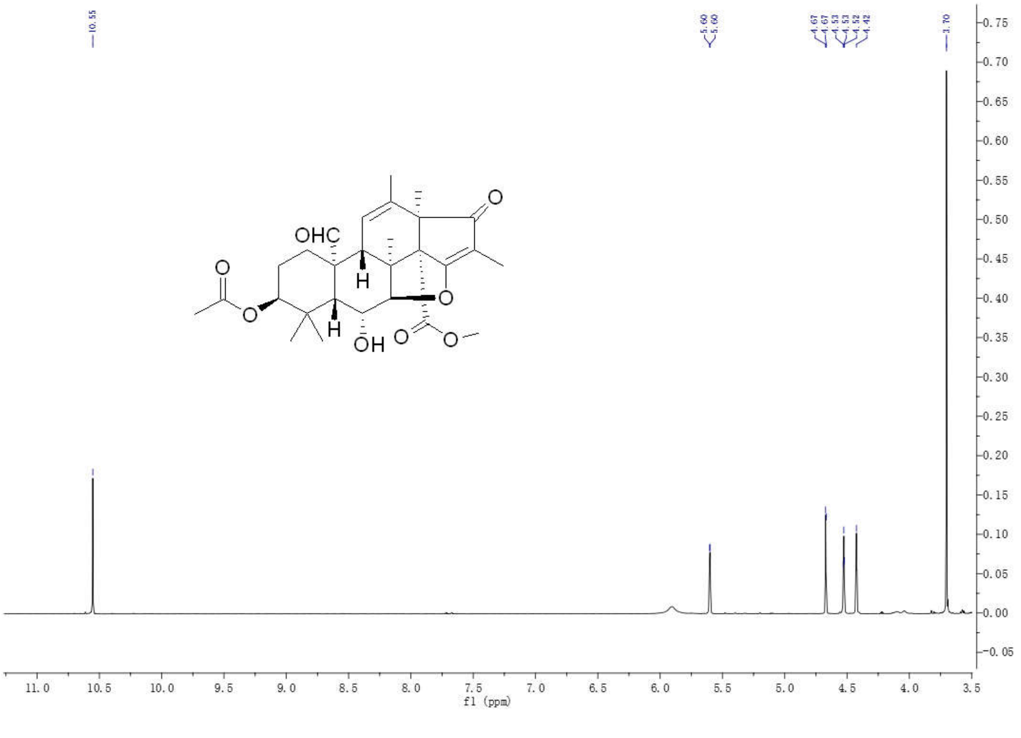

Figure S5. ^{1}H NMR spectrum of andrastone C (**1**)

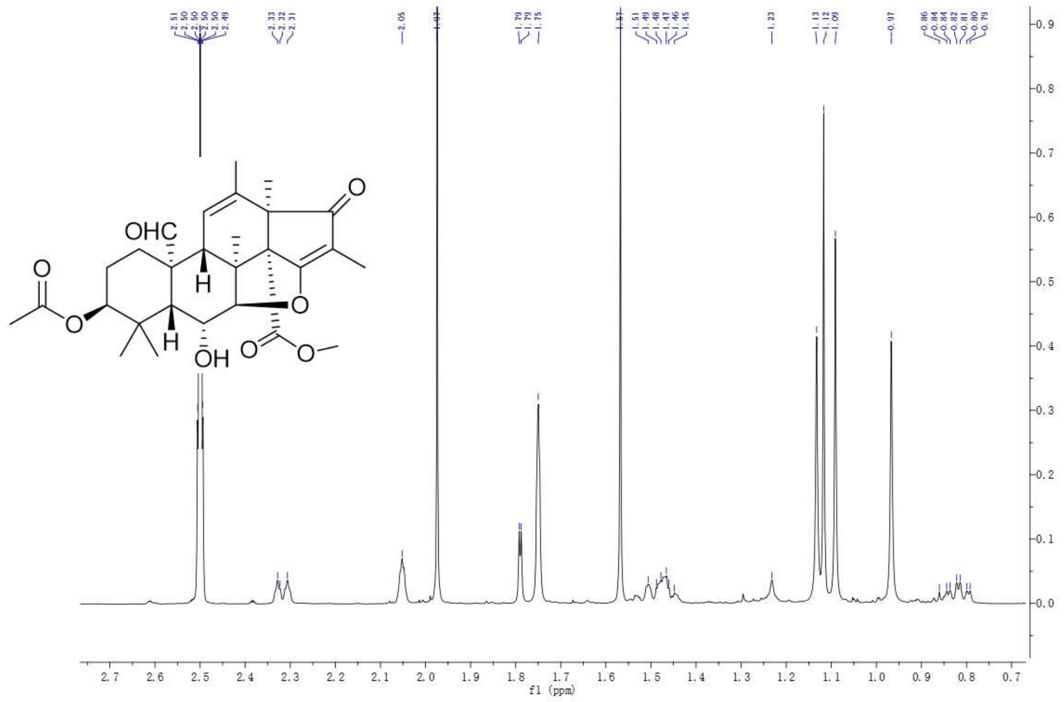

Figure S6. ^{13}C NMR spectrum of andrastone C (**1**)

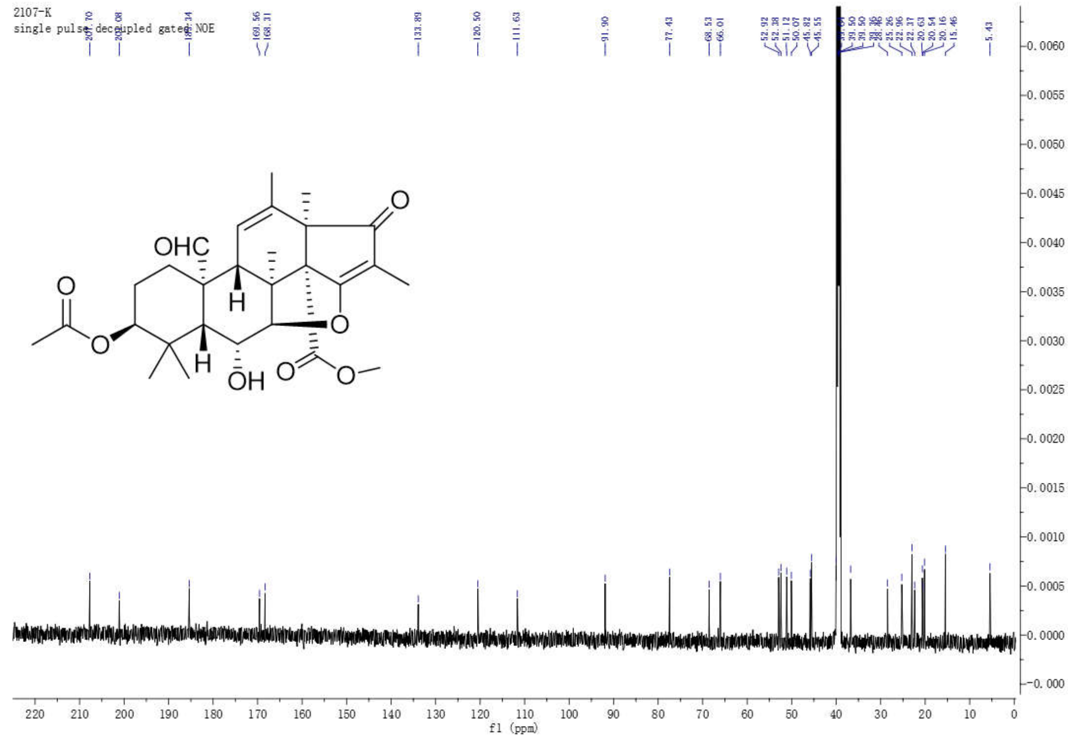

Figure S7. ^{13}C NMR spectrum of andrastone C (**1**)

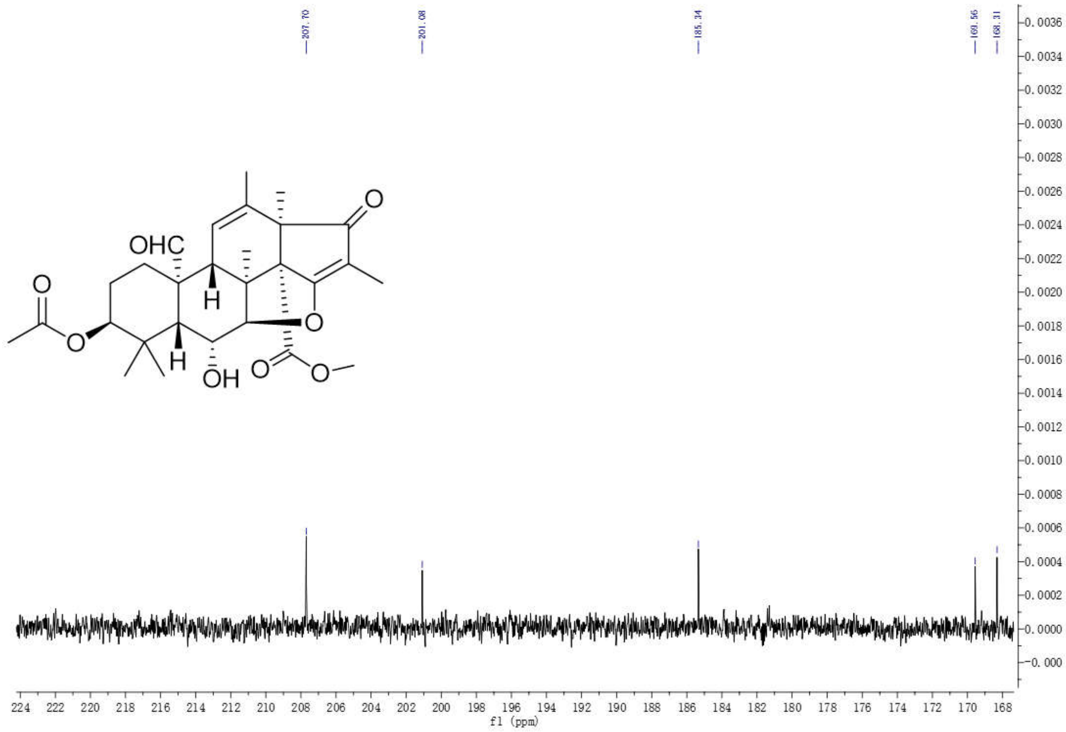

Figure S8. ^{13}C NMR spectrum of andrastone C (**1**)

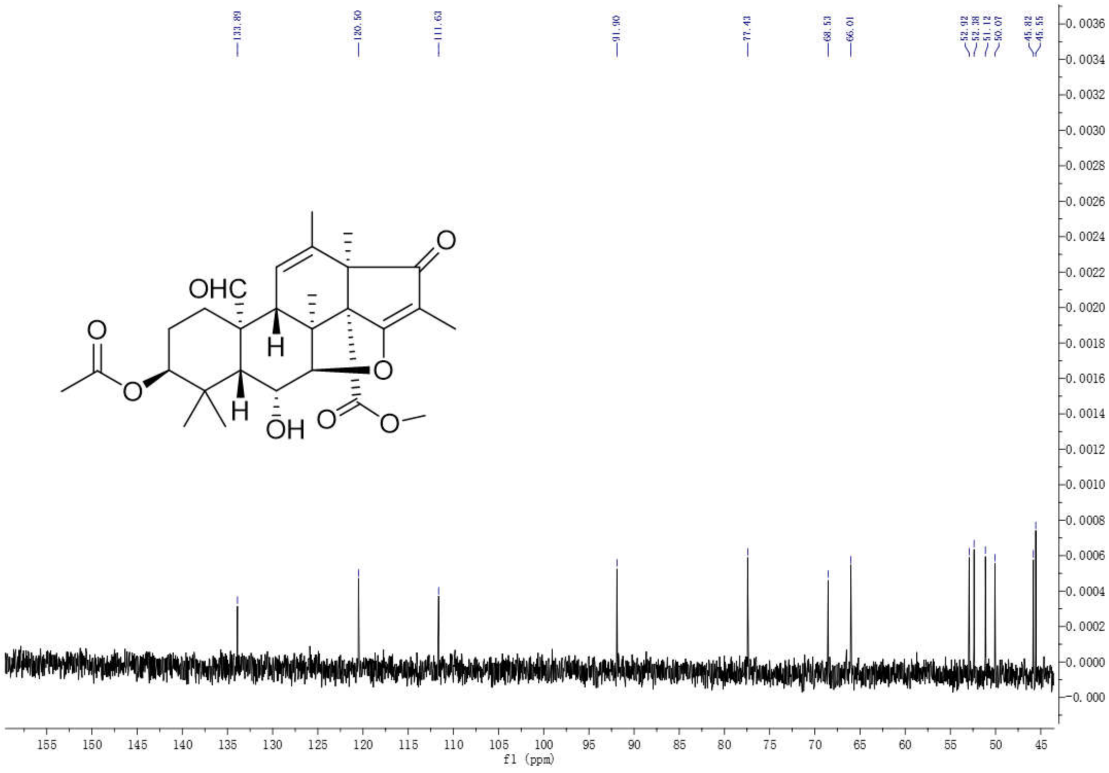

Figure S9. ^{13}C NMR spectrum of andrastone C (**1**)

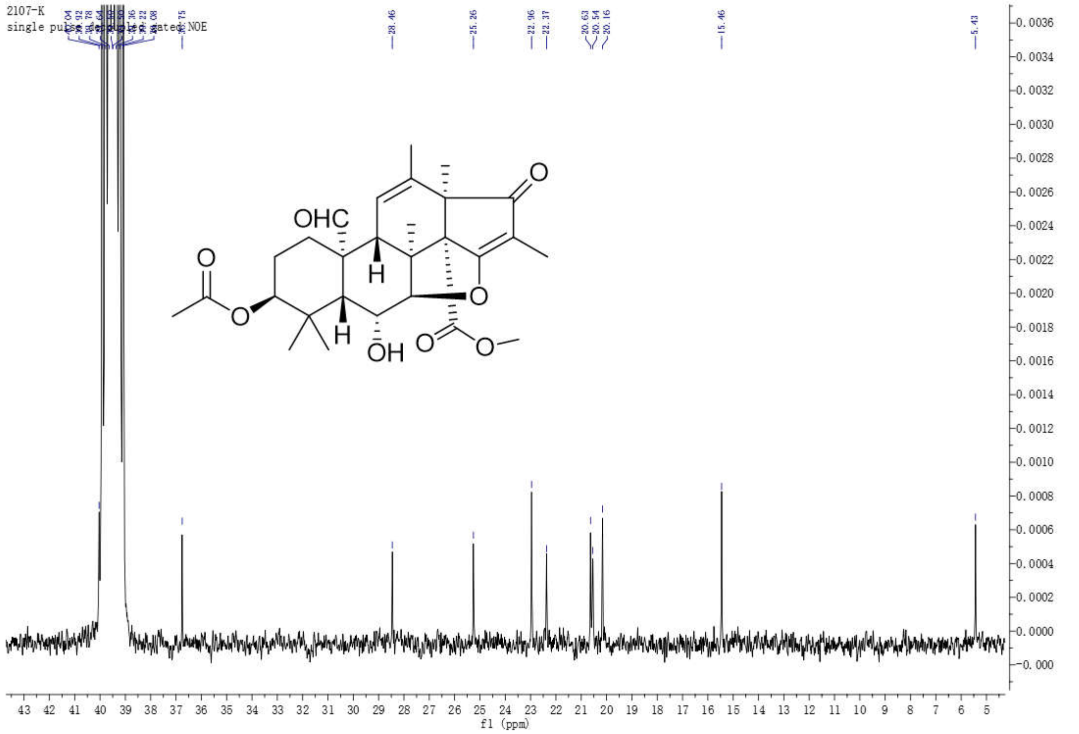

Figure S10. DEPT spectrum of andrastone C (**1**)

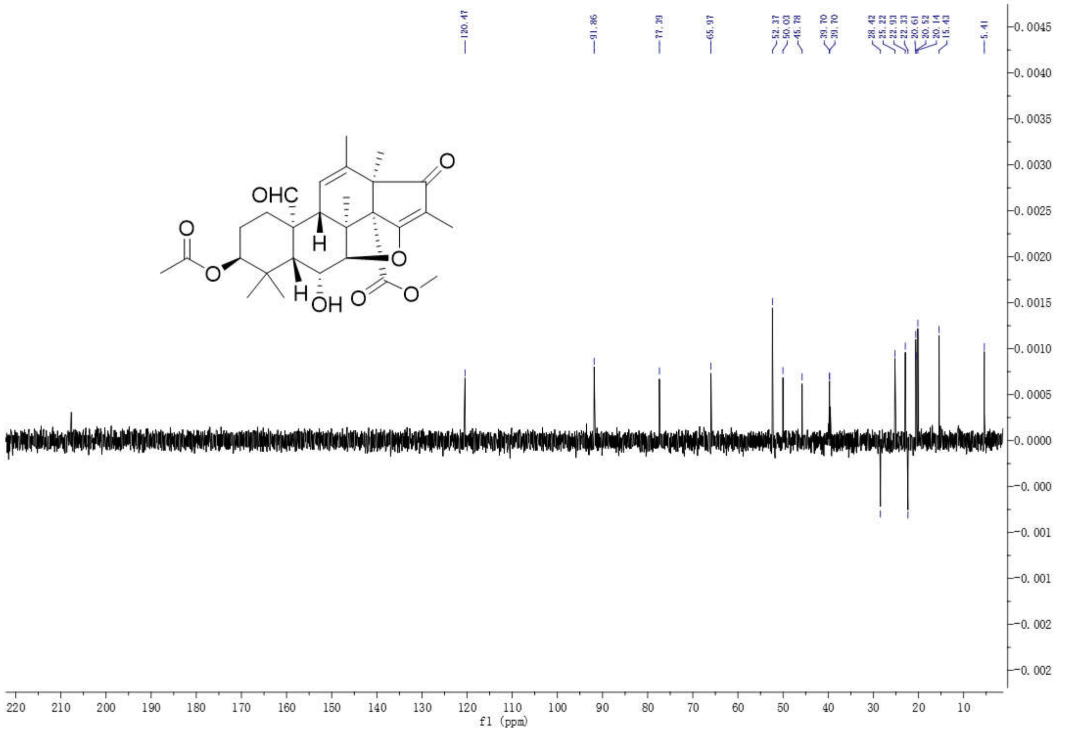

Figure S11. DEPT spectrum of andrastone C (**1**)

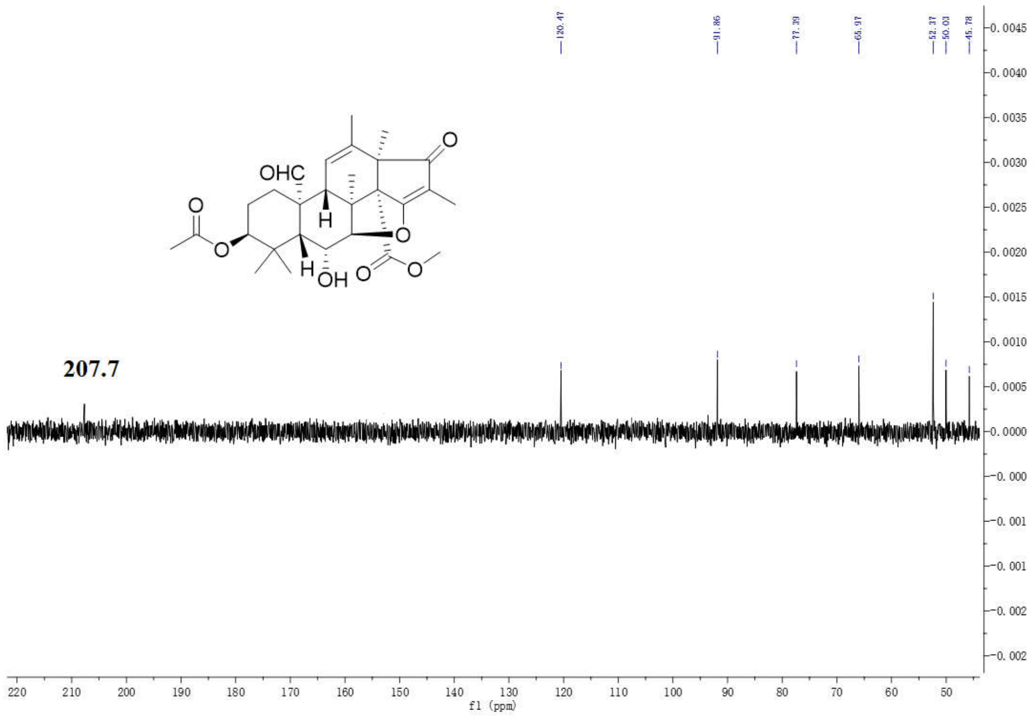

Figure S12. DEPT spectrum of andrastone C (**1**)

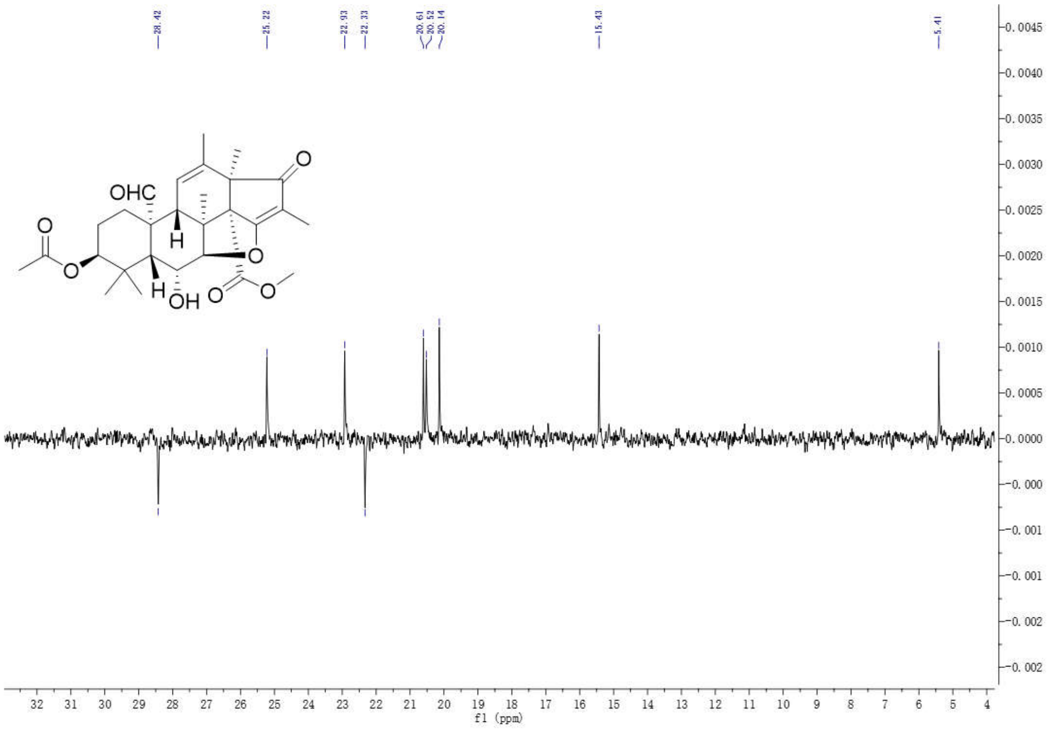

Figure S13. HMQC spectrum of andrastone C (**1**)

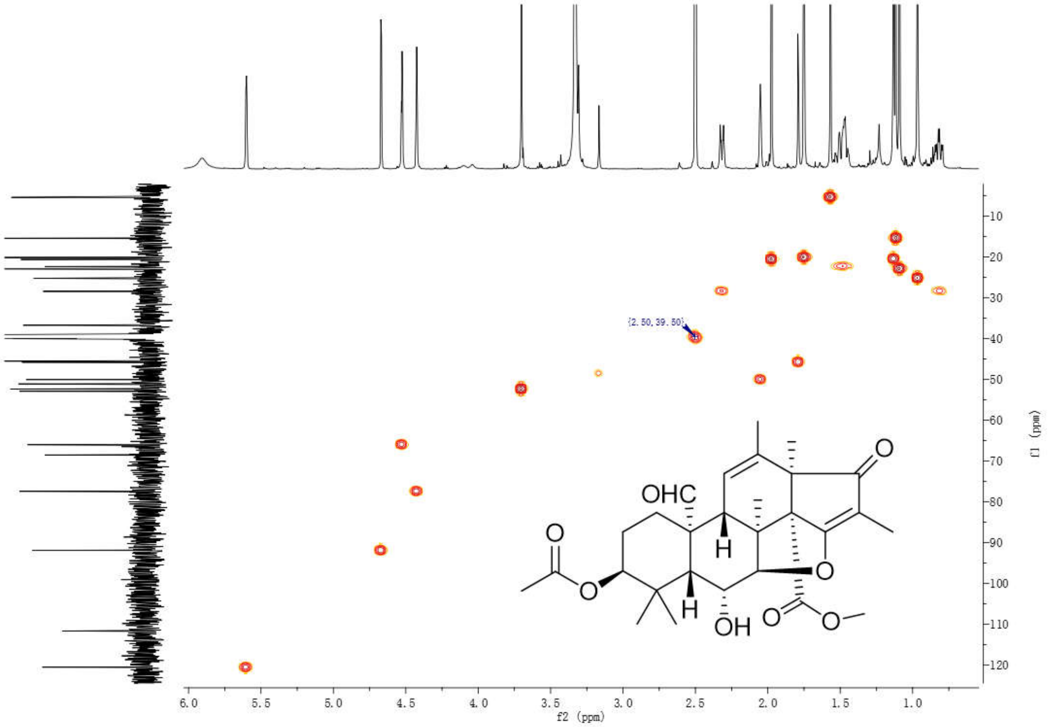

Figure S14. HMQC spectrum of andrastone C (**1**)

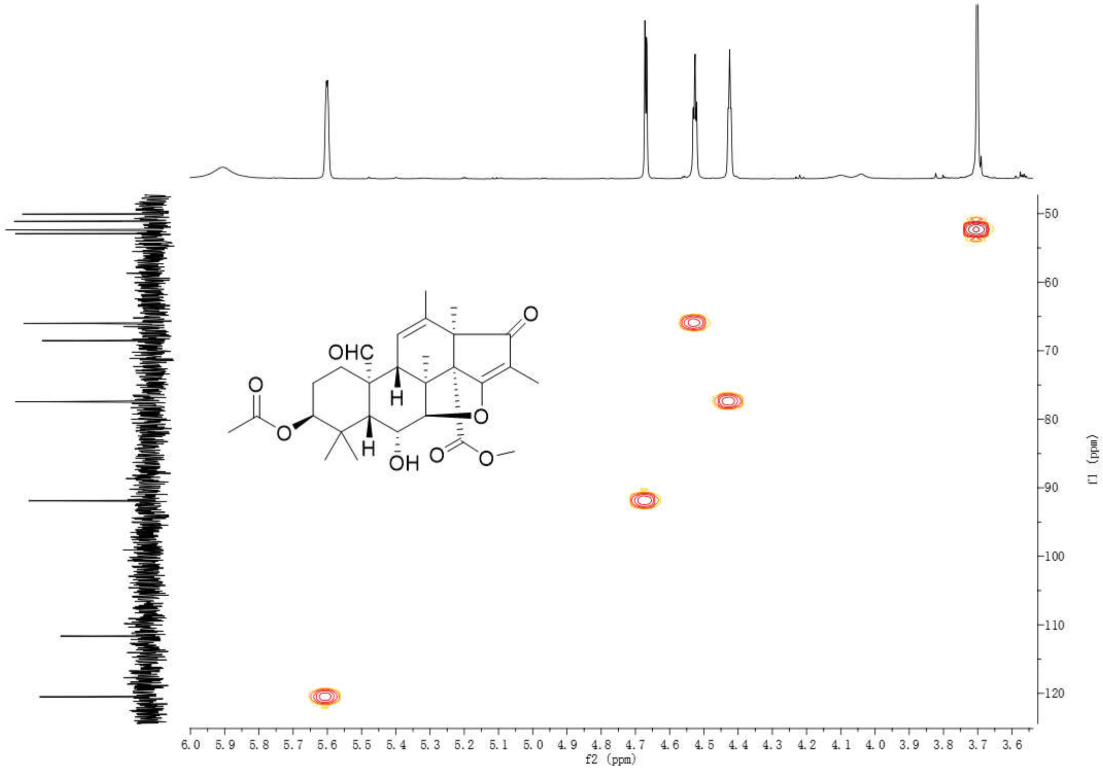

Figure S_{15}. HMQC spectrum of andrastone C (**1**)

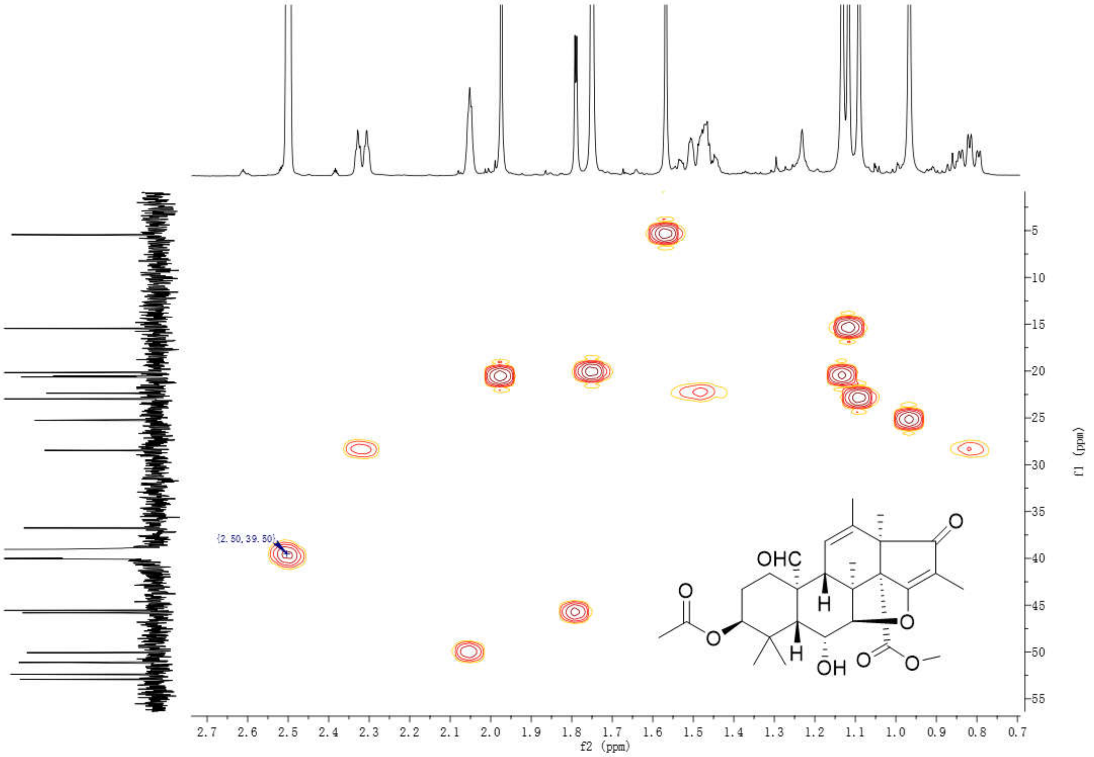

Figure S_{16}. COSY spectrum of andrastone C (**1**)

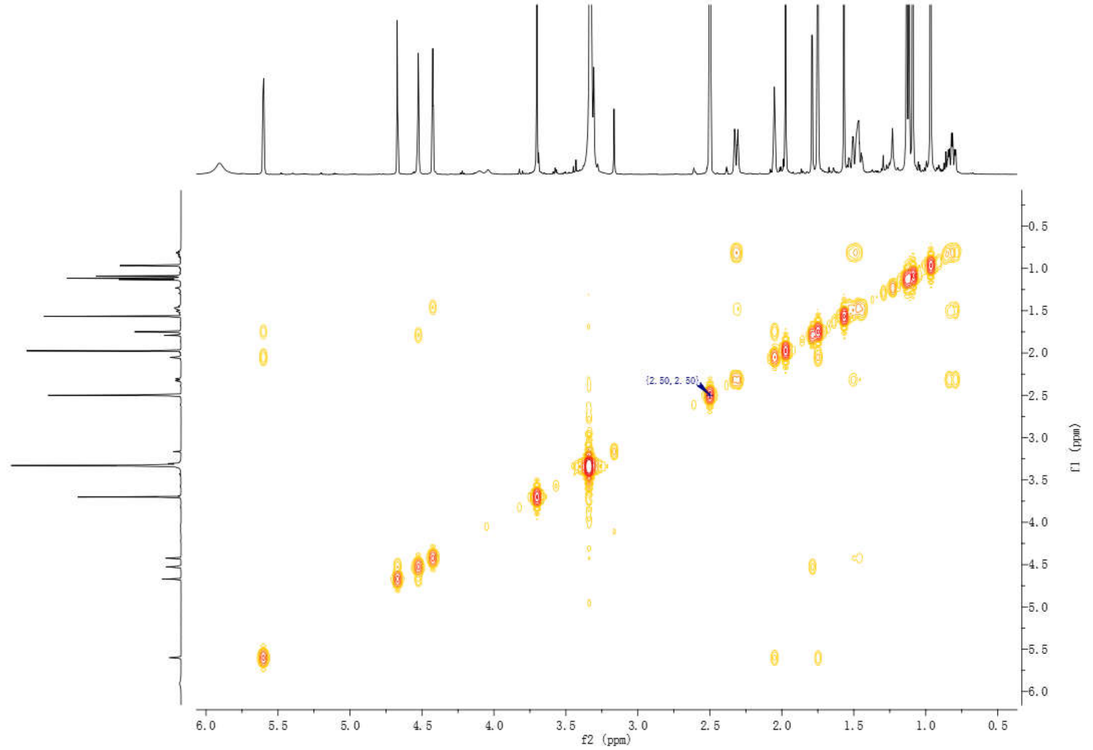

Figure S17. HMBC spectrum of andrastone C (**1**)

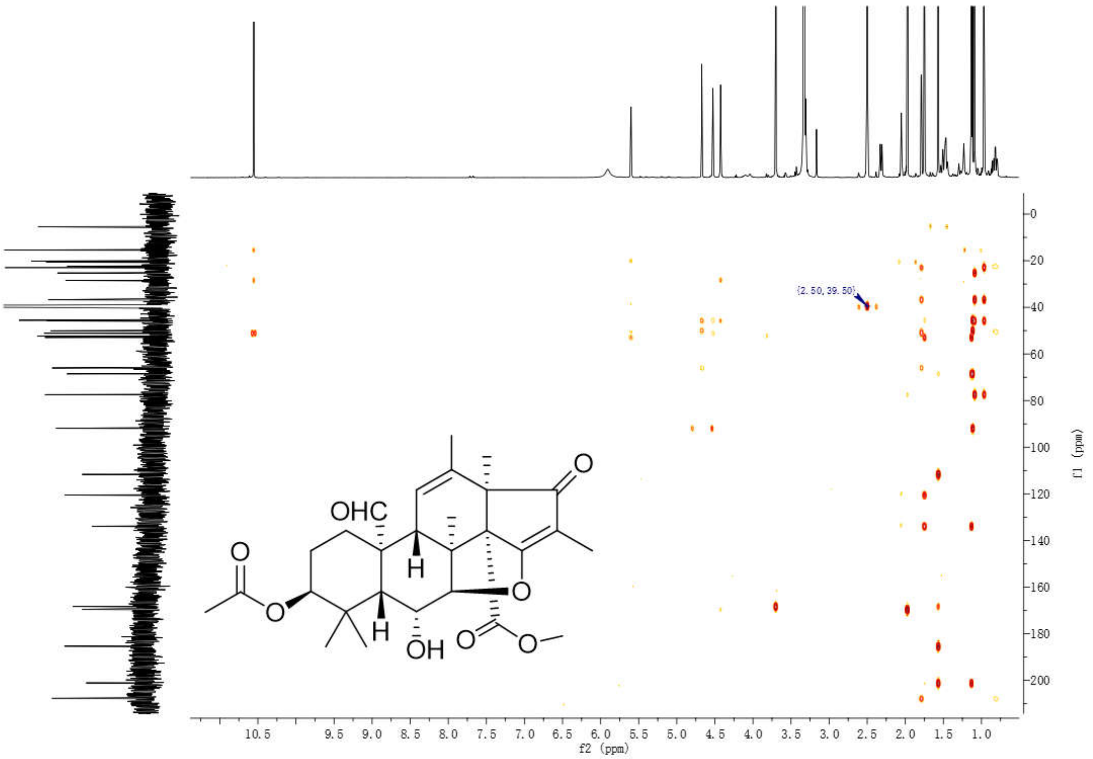

Figure S18. HMBC spectrum of andrastone C (**1**)

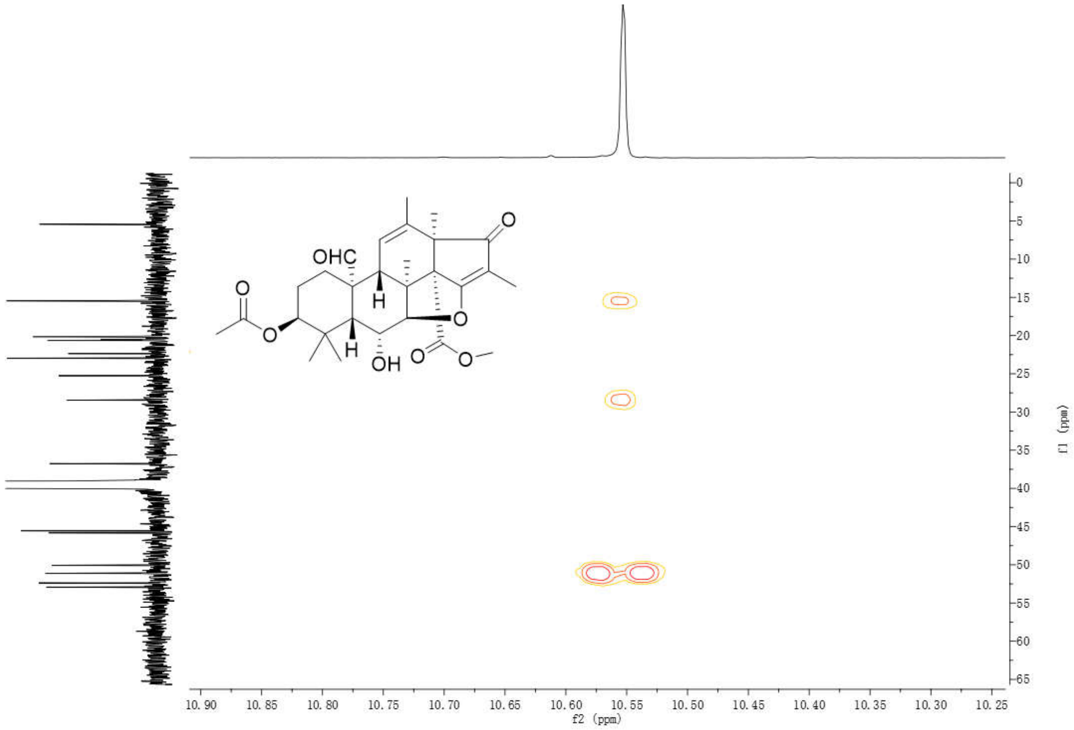

Figure S19. HMBC spectrum of andrastone C (**1**)

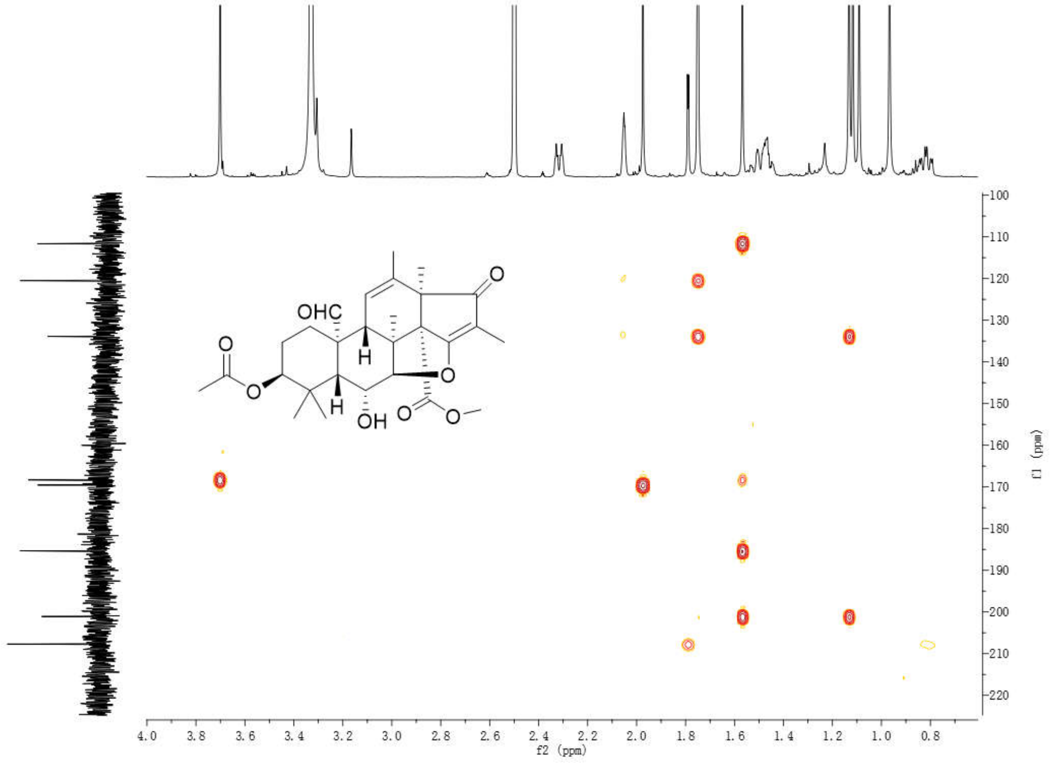

Figure S20. HMBC spectrum of andrastone C (**1**)

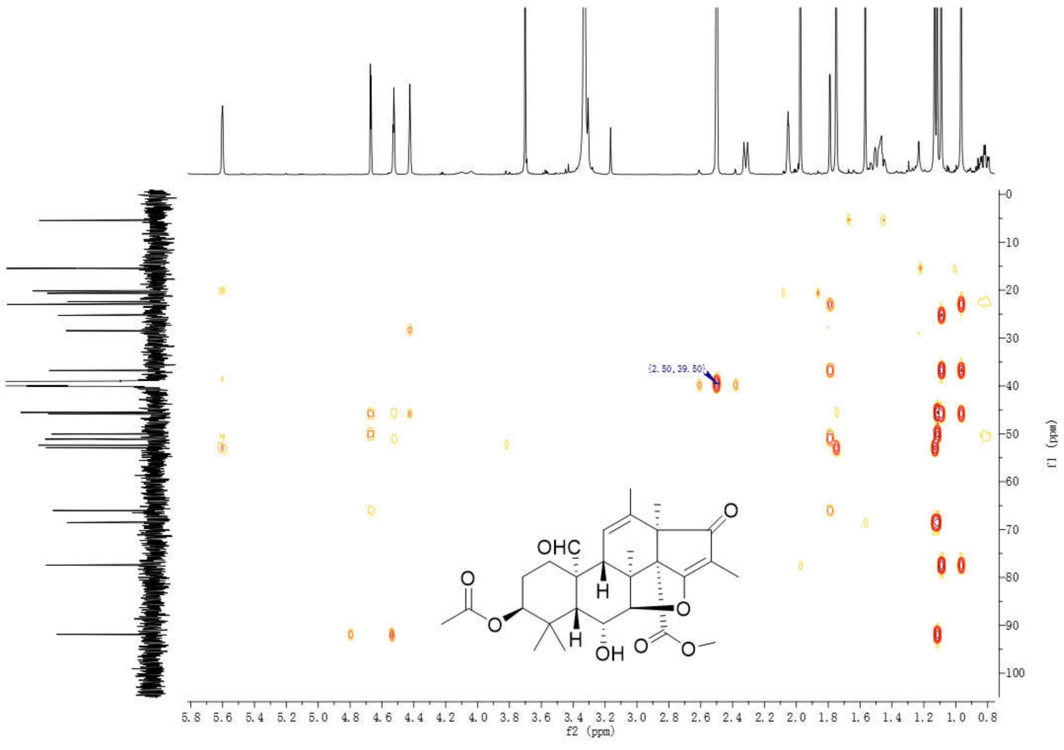

Figure S21. NOESY spectrum of compound andrastone C (**1**)

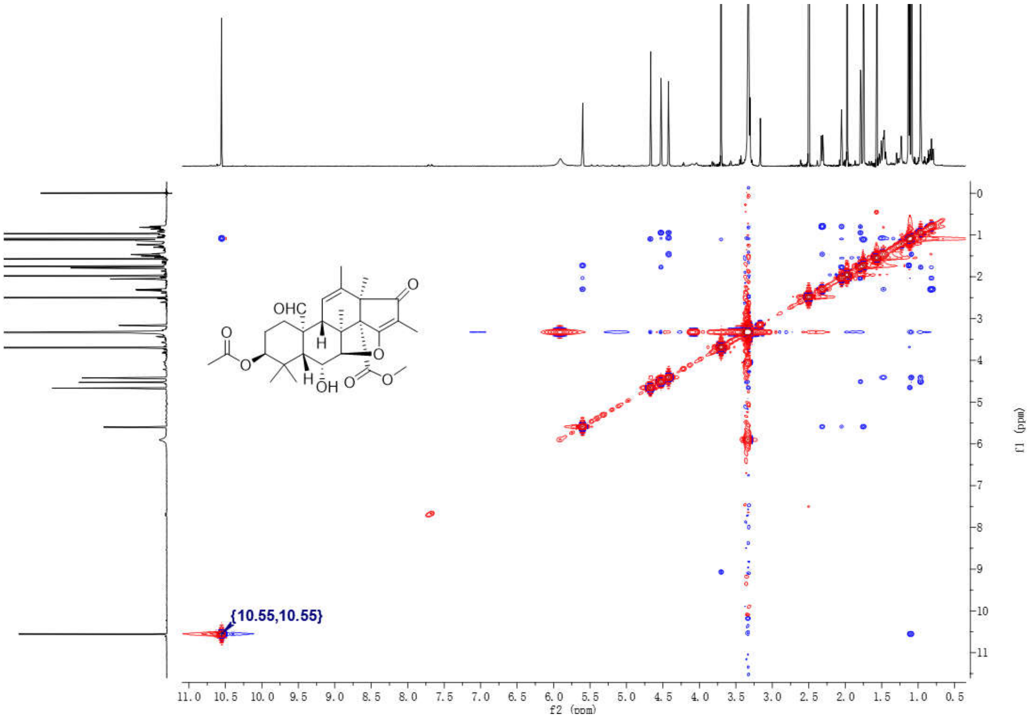

Figure S22. HRESIMS spectrum of andrastone C (**1**)

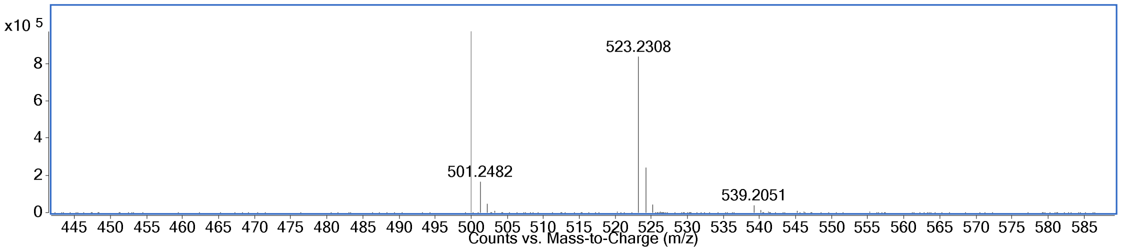

[M+H]$^+$: 501.2482 (calcd for $C_{28}H_{37}O_8$, 501.2488); [M+Na]$^+$: 523.2308 (calcd for $C_{28}H_{36}NaO_8$, 523.2308).

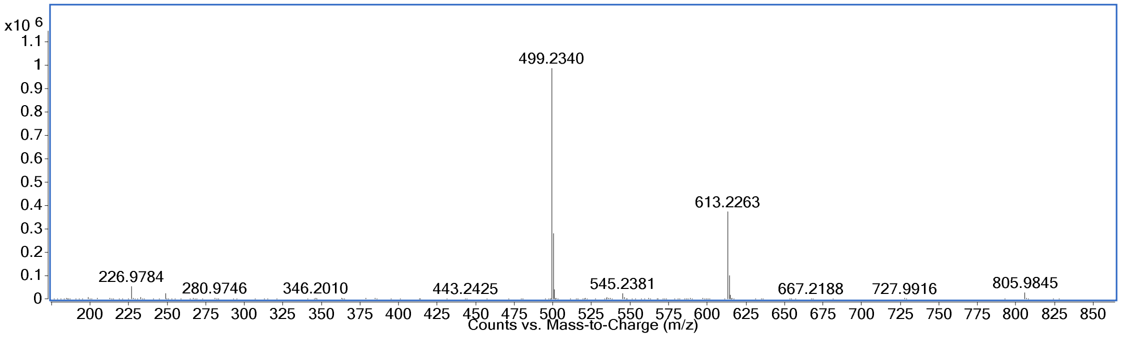

[M–H]$^-$: 499.2340 (calcd for $C_{28}H_{35}O_8$, 499.2332).

Table S8. Crystal data and structure refinement parameters of andrastone C (**1**)

Identification code	2107-K
Empirical formula	$C_{28}H_{36}O_8$
Formula weight	500.57
Temperature/K	293(2)
Crystal system	monoclinic
Space group	$P2_1$
a/Å	8.58899(11)
b/Å	8.86131(11)
c/Å	16.7937(2)
α/°	90
β/°	96.8005(12)
γ/°	90
Volume/Å^3	1269.17(3)
Z	2
ρ_{calc}g/cm^3	1.310
μ/mm^{-1}	0.784
F(000)	536.0
Crystal size/mm^3	0.13 × 0.11 × 0.1
Radiation	Cu Kα (λ = 1.54184)
2Θ range for data collection/°	5.3 to 147.088
Index ranges	$-10 \leq h \leq 10$, $-10 \leq k \leq 10$, $-18 \leq l \leq 20$
Reflections collected	7640
Independent reflections	4316 [R_{int} = 0.0172, R_{sigma} = 0.0240]
Data/restraints/parameters	4316/1/335
Goodness-of-fit on F^2	1.037
Final R indexes [I>=2σ (I)]	R_1 = 0.0311, wR_2 = 0.0856
Final R indexes [all data]	R_1 = 0.0318, wR_2 = 0.0866
Largest diff. peak/hole / e Å^{-3}	0.19/-0.14
Flack/Hooft parameter	-0.02(8)/-0.00(6)
CCDC Number	1979103

Table S9. Fractional atomic coordinates (×10^4) and equivalent isotropic displacement parameters ($Å^2$×10^3) for andrastone C (**1**). U_{eq} is defined as 1/3 of the trace of the orthogonalised U_{IJ}tensor.

Atom	*x*	*y*	*z*	U(eq)
O5	5697.8(16)	6774.5(16)	8055.2(8)	36.4(3)
O8	3171.0(18)	5230(2)	8793.7(10)	48.0(4)
O2	10450(2)	8077(2)	6820.6(9)	52.8(4)
O6	8413(2)	3456(2)	9891.0(11)	61.4(5)
O4	4682(2)	6551(2)	5876.3(9)	53.2(4)
O7	2802.9(19)	3000(2)	8192.1(12)	57.2(5)
O3	7765(3)	2996(2)	5536.2(11)	61.5(5)
O1	12595(2)	8999(3)	6371.0(13)	70.5(6)
C9	7303(2)	4288(2)	7296.6(11)	30.3(4)
C25	3639(2)	4035(3)	8405.8(12)	37.2(4)
C15	7127(2)	5562(3)	9227.1(12)	37.1(4)
C16	6065(2)	5629(2)	8573.1(11)	32.4(4)
C3	9882(3)	7474(3)	6028.7(13)	42.2(5)
C8	5520(2)	4413(2)	7359.9(11)	31.4(4)
C7	5043(2)	6080(2)	7284.9(12)	34.3(4)
C5	7381(2)	6673(2)	6531.0(11)	32.0(4)
C17	5377(2)	4162(2)	8271.8(11)	32.1(4)
C10	7854(2)	4976(2)	6520.2(11)	32.2(4)
C6	5642(2)	6960(3)	6598.7(12)	38.3(4)
C12	7470(3)	2114(2)	8222.7(13)	40.0(5)
C21	7178(3)	4114(3)	5770.2(12)	40.6(5)
C11	7845(2)	2732(2)	7545.6(12)	37.7(4)
C14	7439(2)	3968(3)	9376.0(12)	38.9(5)
C2	10390(3)	5846(3)	5996.6(13)	42.6(5)
C4	8096(3)	7678(3)	5902.5(12)	40.1(5)
C26	11812(3)	8808(3)	6909.9(17)	52.0(6)
C13	6424(2)	2950(2)	8756.0(11)	35.2(4)
C1	9655(2)	4867(3)	6599.9(12)	39.1(4)
C19	7548(3)	7273(3)	5019.9(13)	54.5(6)
C24	8036(3)	6801(3)	9661.1(15)	51.7(6)
C20	4509(3)	3388(3)	6782.0(13)	45.0(5)
C28	1541(3)	5248(4)	8939.8(15)	55.1(7)
C23	5529(3)	1861(3)	9247.5(15)	52.7(6)
C18	7686(4)	9340(3)	6029.5(18)	60.5(7)
C22	8087(4)	587(3)	8491.7(17)	65.2(8)
C27	12264(4)	9299(4)	7757(2)	76.5(9)

Table S_{10}. Anisotropic displacement parameters ($Å^2×10^3$) for andrastone C (**1**). The anisotropic displacement factor exponent takes the form: $-2\pi^2[h^2a^{*2}U_{11}+2hka^*b^*U_{12}+…]$.

Atom	U_{11}	U_{22}	U_{33}	U_{23}	U_{13}	U_{12}
O5	40.0(7)	32.5(7)	37.4(7)	-2.4(6)	6.7(6)	1.9(6)
O8	36.7(8)	58.8(10)	50.8(9)	-11.6(8)	14.6(7)	-1.6(7)
O2	55.3(9)	60.2(11)	43.7(8)	-10.2(8)	9.9(7)	-21.3(8)
O6	60.6(11)	68.3(12)	50.0(10)	4.9(9)	-15.4(8)	7.0(9)
O4	44.3(9)	71.3(12)	41.1(8)	9.5(8)	-6.6(6)	11.5(8)
O7	41.1(8)	57.6(11)	74.5(11)	-13.3(9)	14.0(8)	-14.7(8)
O3	97.0(14)	41.1(10)	48.3(9)	-11.9(8)	17.3(9)	-0.3(9)
O1	47.4(10)	77.7(14)	88.7(14)	-4.9(12)	17.4(10)	-10.1(10)
C9	35.3(9)	30.2(9)	25.9(8)	-0.2(7)	5.5(7)	3.2(7)
C25	33.6(9)	44.8(12)	33.8(9)	1.6(8)	6.6(8)	-3.2(9)
C15	32.3(9)	46.6(12)	33.4(9)	-7.2(9)	7.8(7)	-3.0(9)
C16	31.0(9)	35.6(10)	31.8(9)	-2.8(8)	9.0(7)	0.1(8)
C3	46.3(11)	47.6(13)	34.2(10)	-0.9(9)	10.9(9)	-11.5(10)
C8	31.6(9)	33.2(10)	29.2(9)	-0.3(7)	2.9(7)	-1.5(7)
C7	30.6(9)	38.1(10)	34.2(9)	0.3(8)	3.5(7)	3.2(8)
C5	36.6(10)	31.0(9)	28.7(9)	1.9(8)	5.4(7)	2.0(8)
C17	31.4(9)	34.1(10)	31.1(9)	-0.8(8)	4.8(7)	-2.4(8)
C10	36.6(10)	32.2(10)	28.3(9)	0.1(8)	6.2(7)	1.8(8)
C6	40.1(11)	36.8(11)	38.0(10)	5.9(8)	4.6(8)	5.9(9)
C12	47.9(11)	33.5(10)	38.8(10)	4.9(9)	6.5(9)	5.4(9)
C21	53.7(12)	39.1(11)	30.1(9)	-1.2(8)	9.8(8)	-7.8(9)
C11	45.8(11)	32.7(10)	36.1(10)	-0.1(8)	11.0(8)	7.6(9)
C14	35.5(10)	50.4(13)	31.3(9)	1.2(9)	5.7(8)	0.5(9)
C2	39.5(11)	52.0(13)	38.2(10)	-1.9(10)	12.8(9)	-2.3(10)
C4	48.2(11)	38.1(11)	34.8(10)	7.3(9)	8.5(8)	-0.9(9)
C26	43.8(12)	39.6(13)	72.1(16)	-6.7(12)	5.1(11)	0.7(10)
C13	36.3(10)	37.8(11)	32.0(9)	4.9(8)	5.9(7)	-1.2(8)
C1	37.6(10)	43.7(11)	37.4(10)	3.1(9)	9.9(8)	5.5(9)
C19	62.3(15)	66.4(17)	34.1(11)	14.1(11)	3.3(10)	-4.0(13)
C24	44.4(12)	59.9(15)	50.5(13)	-16.6(12)	4.4(10)	-7.7(11)
C20	45.9(12)	49.6(13)	38.1(10)	-5.3(10)	-0.2(9)	-10.8(10)
C28	35.5(11)	78.4(19)	53.5(13)	-5.3(13)	14.2(10)	6.1(11)
C23	50.3(13)	56.6(15)	52.5(13)	20.1(12)	11.9(10)	-5.0(11)
C18	78.7(18)	36.8(13)	70.1(17)	15.2(12)	25.9(14)	1.5(12)
C22	91(2)	48.7(15)	59.5(15)	19.2(12)	23.5(14)	25.5(14)
C27	65.5(17)	72(2)	89(2)	-35.6(18)	-4.7(15)	-6.1(15)

Table S_{11}. Bond lengths for andrastone C (**1**).

Atom	Atom	Length/Å	Atom	Atom	Length/Å
O5	C16	1.349(2)	C3	C4	1.534(3)
O5	C7	1.482(2)	C8	C7	1.534(3)
O8	C25	1.330(3)	C8	C17	1.567(2)
O8	C28	1.449(3)	C8	C20	1.523(3)
O2	C3	1.463(3)	C7	C6	1.530(3)
O2	C26	1.330(3)	C5	C10	1.559(3)
O6	C14	1.218(3)	C5	C6	1.533(3)
O4	C6	1.431(3)	C5	C4	1.562(3)
O7	C25	1.194(3)	C17	C13	1.565(3)
O3	C21	1.199(3)	C10	C21	1.527(3)
O1	C26	1.202(3)	C10	C1	1.540(3)
C9	C8	1.551(3)	C12	C11	1.335(3)
C9	C10	1.562(2)	C12	C13	1.533(3)
C9	C11	1.499(3)	C12	C22	1.503(3)
C25	C17	1.540(3)	C14	C13	1.563(3)
C15	C16	1.343(3)	C2	C1	1.526(3)
C15	C14	1.454(3)	C4	C19	1.543(3)
C15	C24	1.487(3)	C4	C18	1.535(3)
C16	C17	1.492(3)	C26	C27	1.494(4)
C3	C2	1.510(3)	C13	C23	1.534(3)

Table S_{12}. Bond angles for andrastone C (**1**).

Atom	Atom	Atom	Angle/°	Atom	Atom	Atom	Angle/°
C16	O5	C7	106.63(15)	C13	C17	C8	119.92(15)
C25	O8	C28	116.49(18)	C5	C10	C9	105.14(14)
C26	O2	C3	118.36(18)	C21	C10	C9	111.72(16)
C8	C9	C10	115.37(15)	C21	C10	C5	114.68(17)
C11	C9	C8	108.84(16)	C21	C10	C1	108.69(16)
C11	C9	C10	118.46(15)	C1	C10	C9	107.68(15)
O8	C25	C17	111.62(17)	C1	C10	C5	108.67(16)
O7	C25	O8	123.53(18)	O4	C6	C7	107.34(18)
O7	C25	C17	124.85(19)	O4	C6	C5	111.34(16)
C16	C15	C14	106.11(18)	C7	C6	C5	112.86(16)
C16	C15	C24	129.4(2)	C11	C12	C13	121.09(18)
C14	C15	C24	123.9(2)	C11	C12	C22	120.9(2)
O5	C16	C17	112.58(16)	C22	C12	C13	118.03(19)
C15	C16	O5	130.15(19)	O3	C21	C10	123.4(2)
C15	C16	C17	116.22(18)	C12	C11	C9	121.11(18)
O2	C3	C2	108.36(19)	O6	C14	C15	125.6(2)
O2	C3	C4	107.65(17)	O6	C14	C13	122.7(2)
C2	C3	C4	113.33(18)	C15	C14	C13	111.66(17)
C9	C8	C17	104.41(14)	C3	C2	C1	112.07(17)
C7	C8	C9	108.62(15)	C3	C4	C5	107.84(17)
C7	C8	C17	99.56(15)	C3	C4	C19	106.93(17)
C20	C8	C9	113.97(16)	C3	C4	C18	109.6(2)
C20	C8	C7	113.29(17)	C19	C4	C5	114.64(19)
C20	C8	C17	115.69(17)	C18	C4	C5	109.78(18)
O5	C7	C8	104.95(15)	C18	C4	C19	107.9(2)
O5	C7	C6	108.61(16)	O2	C26	C27	112.1(2)
C6	C7	C8	116.25(16)	O1	C26	O2	123.2(2)
C10	C5	C4	114.74(15)	O1	C26	C27	124.7(3)
C6	C5	C10	114.78(17)	C12	C13	C17	111.63(15)
C6	C5	C4	114.84(17)	C12	C13	C14	110.44(16)
C25	C17	C8	110.03(15)	C12	C13	C23	112.08(19)
C25	C17	C13	112.43(16)	C14	C13	C17	101.02(16)
C16	C17	C25	111.46(16)	C23	C13	C17	114.66(17)
C16	C17	C8	97.69(15)	C23	C13	C14	106.27(17)
C16	C17	C13	103.98(15)	C2	C1	C10	113.37(17)

Table S13. Torsion angles for andrastone C (**1**).

A	B	C	D	Angle/°	A	B	C	D	Angle/°
O5	C16	C17	C25	-80.06(19)	C5	C10	C1	C2	-51.7(2)
O5	C16	C17	C8	35.06(18)	C17	C8	C7	O5	35.91(17)
O5	C16	C17	C13	158.59(14)	C17	C8	C7	C6	155.92(17)
O5	C7	C6	O4	-164.47(15)	C10	C9	C8	C7	-56.2(2)
O5	C7	C6	C5	72.5(2)	C10	C9	C8	C17	-161.73(16)
O8	C25	C17	C16	-3.0(2)	C10	C9	C8	C20	71.1(2)
O8	C25	C17	C8	-110.28(19)	C10	C9	C11	C12	176.9(2)
O8	C25	C17	C13	113.3(2)	C10	C5	C6	O4	-70.5(2)
O2	C3	C2	C1	63.4(2)	C10	C5	C6	C7	50.3(2)
O2	C3	C4	C5	-65.0(2)	C10	C5	C4	C3	-54.8(2)
O2	C3	C4	C19	171.24(18)	C10	C5	C4	C19	64.1(2)
O2	C3	C4	C18	54.5(2)	C10	C5	C4	C18	-174.2(2)
O6	C14	C13	C17	178.49(19)	C6	C5	C10	C9	-55.2(2)
O6	C14	C13	C12	-63.3(3)	C6	C5	C10	C21	68.0(2)
O6	C14	C13	C23	58.5(3)	C6	C5	C10	C1	-170.21(15)
O7	C25	C17	C16	177.0(2)	C6	C5	C4	C3	168.98(17)
O7	C25	C17	C8	69.8(3)	C6	C5	C4	C19	-72.1(2)
O7	C25	C17	C13	-66.7(3)	C6	C5	C4	C18	49.6(3)
C9	C8	C7	O5	-72.93(17)	C21	C10	C1	C2	73.7(2)
C9	C8	C7	C6	47.1(2)	C11	C9	C8	C7	167.81(16)
C9	C8	C17	C25	-172.12(16)	C11	C9	C8	C17	62.29(19)
C9	C8	C17	C16	71.64(17)	C11	C9	C8	C20	-64.9(2)
C9	C8	C17	C13	-39.4(2)	C11	C9	C10	C5	-168.91(16)
C9	C10	C21	O3	-84.7(2)	C11	C9	C10	C21	66.1(2)
C9	C10	C1	C2	-165.08(17)	C11	C9	C10	C1	-53.2(2)
C25	C17	C13	C12	130.23(18)	C11	C12	C13	C17	23.1(3)
C25	C17	C13	C14	-112.39(17)	C11	C12	C13	C14	-88.5(3)
C25	C17	C13	C23	1.4(3)	C11	C12	C13	C23	153.2(2)
C15	C16	C17	C25	110.45(19)	C14	C15	C16	O5	-159.30(18)
C15	C16	C17	C8	-134.43(17)	C14	C15	C16	C17	8.0(2)
C15	C16	C17	C13	-10.9(2)	C2	C3	C4	C5	54.8(2)
C15	C14	C13	C17	-4.6(2)	C2	C3	C4	C19	-68.9(2)
C15	C14	C13	C12	113.65(18)	C2	C3	C4	C18	174.29(19)
C15	C14	C13	C23	-124.57(19)	C4	C3	C2	C1	-56.1(2)
C16	O5	C7	C8	-15.83(18)	C4	C5	C10	C9	168.61(16)
C16	O5	C7	C6	-140.79(16)	C4	C5	C10	C21	-68.3(2)
C16	C15	C14	O6	175.2(2)	C4	C5	C10	C1	53.6(2)

C16	C15	C14	C13	-1.6(2)	C4	C5	C6	O4	65.7(2)
C16	C17	C13	C12	-109.07(18)	C4	C5	C6	C7	-173.52(18)
C16	C17	C13	C14	8.31(17)	C26	O2	C3	C2	94.5(2)
C16	C17	C13	C23	122.1(2)	C26	O2	C3	C4	-142.5(2)
C3	O2	C26	O1	0.1(4)	C13	C12	C11	C9	2.2(3)
C3	O2	C26	C27	-177.7(2)	C1	C10	C21	O3	34.0(3)
C3	C2	C1	C10	54.2(3)	C24	C15	C16	O5	11.5(3)
C8	C9	C10	C5	59.5(2)	C24	C15	C16	C17	178.82(19)
C8	C9	C10	C21	-65.5(2)	C24	C15	C14	O6	3.8(3)
C8	C9	C10	C1	175.25(17)	C24	C15	C14	C13	-173.04(18)
C8	C9	C11	C12	-48.7(3)	C20	C8	C7	O5	159.35(15)
C8	C7	C6	O4	77.5(2)	C20	C8	C7	C6	-80.6(2)
C8	C7	C6	C5	-45.5(2)	C20	C8	C17	C25	-46.0(2)
C8	C17	C13	C12	-1.5(3)	C20	C8	C17	C16	-162.27(18)
C8	C17	C13	C14	115.92(18)	C20	C8	C17	C13	86.7(2)
C8	C17	C13	C23	-130.3(2)	C28	O8	C25	O7	-1.2(3)
C7	O5	C16	C15	154.60(19)	C28	O8	C25	C17	178.83(18)
C7	O5	C16	C17	-13.03(19)	C22	C12	C11	C9	-176.7(2)
C7	C8	C17	C25	75.69(19)	C22	C12	C13	C17	-158.0(2)
C7	C8	C17	C16	-40.55(16)	C22	C12	C13	C14	90.4(3)
C7	C8	C17	C13	-151.59(17)	C22	C12	C13	C23	-27.9(3)
C5	C10	C21	O3	155.78(19)					

Table S14. Hydrogen atom coordinates (Å×10^4) and isotropic displacement parameters (Å^2×10^3) for andrastone C (**1**).

Atom	*x*	*y*	*z*	U(eq)
H4	3946.01	7143.07	5795.54	80
H9	7787.97	4938.68	7728.48	36
H3	10349.58	8049.3	5618.92	51
H7	3896.6	6150.76	7233.23	41
H5	7906.92	7034.3	7045.31	38
H6	5499.68	8040.22	6693.81	46
H21	6264.25	4475.87	5480.3	49
H11	8451.83	2187.48	7223.01	45
H2A	11522.48	5791.1	6106.87	51
H2B	10092.02	5455.45	5460.62	51
H1A	10076.42	5167.89	7137.82	47
H1B	9950.62	3823.67	6527.5	47
H19A	8038.43	7938.26	4673.53	82
H19B	6430.13	7377.9	4917.99	82
H19C	7836	6249.29	4919.35	82
H24A	9045.35	6878.71	9472.22	78
H24B	8169.9	6587.95	10225.55	78
H24C	7480.57	7735.71	9565.68	78
H20A	4625.02	3670.67	6240.21	67
H20B	3430.62	3488.37	6870.89	67
H20C	4836.02	2359.68	6870.56	67
H28A	883.06	5077.82	8445.05	83
H28B	1296.13	6210.43	9155.8	83
H28C	1365.72	4467.29	9315.43	83
H23A	4859.98	2426.04	9556.86	79
H23B	6262.7	1285.3	9601.36	79
H23C	4904.87	1190.39	8891.92	79
H18A	7876.96	9579.94	6590.01	91
H18B	6600.47	9508.49	5842.07	91
H18C	8325.34	9971.39	5735.75	91
H22A	7226.33	-96.39	8512.94	98
H22B	8668.64	669.5	9014.51	98
H22C	8761.73	213.51	8119.44	98
H27A	12073.56	10361.66	7801.67	115
H27B	13357.34	9096.65	7907.72	115
H27C	11653.01	8755.65	8104.32	115

Table S_{15}. Crystal data and structure refinement parameters of andrastone B (**2**)

Identification code	2017J
Empirical formula	$C_{28}H_{38}O_{10}$
Formula weight	534.58
Temperature/K	293(2)
Crystal system	orthorhombic
Space group	$P2_12_12_1$
a/Å	9.1880(3)
b/Å	13.2582(5)
c/Å	22.8448(6)
α/°	90
β/°	90
γ/°	90
Volume/Å^3	2782.87(16)
Z	4
ρ_{calc}g/cm^3	1.276
μ/mm^{-1}	0.802
F(000)	1144.0
Crystal size/mm^3	0.13 × 0.11 × 0.1
Radiation	Cu Kα (λ = 1.54184)
2Θ range for data collection/°	7.71 to 147.056
Index ranges	$-9 \leq h \leq 11$, $-15 \leq k \leq 16$, $-19 \leq l \leq 27$
Reflections collected	13131
Independent reflections	5414 [R_{int} = 0.0263, R_{sigma} = 0.0313]
Data/restraints/parameters	5414/0/355
Goodness-of-fit on F^2	1.052
Final R indexes [I>=2σ (I)]	R_1 = 0.0438, wR_2 = 0.1126
Final R indexes [all data]	R_1 = 0.0499, wR_2 = 0.1180
Largest diff. peak/hole / e Å^{-3}	0.22/-0.17
Flack/Hooft parameter	-0.01(11)/0.08(10)
CCDC Number	1976943

Table S_{16}. Fractional atomic coordinates (×10^4) and equivalent isotropic displacement parameters ($Å^2$×10^3) for andrastone B (**2**). U_{eq} is defined as 1/3 of the trace of the orthogonalised U_{IJ}tensor.

Atom	*x*	*y*	*z*	U(eq)
O3	10189(2)	3527.8(16)	5501.4(9)	50.5(5)
O8	5171(2)	1916.8(17)	6057.0(8)	52.3(5)
O2	5732(2)	5784.5(18)	5462.8(10)	61.0(6)
O4	11038(2)	4532(2)	6204.7(11)	66.9(7)
O6	6516(3)	-64.8(19)	6676.8(11)	72.6(7)
O5	8499(4)	153(2)	6136.7(15)	91.5(9)
O9	4542(4)	2811(3)	7244.9(11)	90.3(9)
O7	5724(3)	1162(3)	8024.6(10)	94.9(10)
C9	7877(3)	3407(2)	6476.6(10)	40.1(5)
C5	7781(3)	4093.5(19)	5462.0(10)	37.0(5)
C10	8404(3)	4297(2)	6077.8(11)	39.2(5)
C4	7837(3)	4996(2)	5028.4(12)	46.9(6)
C8	8397(3)	2339(2)	6290.9(11)	42.7(6)
C21	10013(3)	4165(2)	5954.3(12)	47.5(6)
C16	5725(3)	1795(2)	6528.0(11)	44.2(6)
C6	8740(3)	3198(2)	5300.8(11)	42.6(6)
C17	7379(3)	1601(2)	6631.9(11)	46.1(6)
O10	4002(3)	4099(3)	6368(2)	103.4(11)
C3	7269(3)	5944(2)	5333.8(14)	53.1(7)
C7	8297(3)	2233(2)	5611.9(11)	44.5(6)
C1	8000(3)	5321(2)	6337.9(13)	49.4(7)
C25	7586(4)	491(2)	6443.2(14)	60.2(8)
C2	8055(4)	6184(2)	5900.1(15)	59.3(8)
C13	7551(4)	1694(3)	7322.8(12)	57.5(8)
C12	7932(4)	2779(3)	7505.2(12)	63.1(9)
C11	8088(3)	3523(3)	7127.3(12)	56.8(7)
C14	6011(4)	1485(3)	7545.9(13)	64.2(9)
C15	4874(4)	1797(3)	7096.4(12)	58.7(8)
C18	9371(4)	5241(3)	4787.5(15)	62.2(8)
C20	9996(3)	2121(3)	6480.0(15)	59.6(8)
C26	4787(4)	6486(3)	5303(2)	79.1(11)
C19	6882(4)	4729(3)	4502.6(14)	66.0(9)
O1	5155(4)	7261(3)	5100(3)	183(3)
C24	3550(4)	1134(4)	7085.3(17)	90.7(15)
C28	6587(7)	-1145(3)	6548(2)	96.2(15)
C23	8607(5)	930(4)	7605.3(17)	86.9(13)
C27	3259(4)	6175(3)	5404(2)	84.3(12)
C22	8113(7)	2969(4)	8157.4(14)	104.2(17)

Table S_{17}. Anisotropic displacement parameters (Å^2×10^3) for andrastone B (**2**). The anisotropic displacement factor exponent takes the form: $-2\pi^2[h^2a^{*2}U_{11}+2hka^*b^*U_{12}+\ldots]$.

Atom	U_{11}	U_{22}	U_{33}	U_{23}	U_{13}	U_{12}
O3	39.5(9)	55.5(11)	56.6(10)	-8.3(9)	9.5(8)	2.1(9)
O8	52.2(11)	63.8(13)	40.8(9)	5.4(9)	-7.0(8)	-5.2(10)
O2	48.3(11)	54.4(12)	80.4(14)	16.4(11)	-1.7(11)	4.6(10)
O4	38.1(11)	81.6(17)	81.1(15)	-17.4(13)	-11.3(10)	-6.5(10)
O6	86.1(17)	51.7(13)	79.9(15)	15.0(11)	15.7(13)	2.8(13)
O5	100(2)	52.8(14)	122(2)	4.5(15)	44.3(19)	18.5(15)
O9	100(2)	112(2)	58.7(13)	-9.2(14)	3.6(14)	46.3(19)
O7	90.1(19)	144(3)	50.4(12)	40.4(15)	6.2(13)	1(2)
C9	35.7(12)	49.2(15)	35.6(11)	-4.6(10)	-0.3(10)	4.0(11)
C5	37.6(12)	37.4(12)	36.1(10)	-3.7(9)	0.1(10)	-5.1(10)
C10	34.9(12)	42.3(13)	40.4(11)	-8.3(10)	-2.0(10)	0.7(10)
C4	50.2(15)	43.0(14)	47.4(13)	3.7(11)	-1.1(12)	-8.6(12)
C8	38.0(13)	47.7(14)	42.2(12)	1.1(11)	0.5(11)	8.9(11)
C21	38.1(13)	52.9(16)	51.5(14)	-7.2(12)	-1.5(12)	-1.3(12)
C16	49.3(14)	43.7(14)	39.5(12)	4.5(11)	-0.9(11)	1.9(12)
C6	45.3(13)	44.1(14)	38.5(11)	-6.8(11)	5.7(10)	-1.6(11)
C17	46.6(14)	49.8(16)	42.0(12)	7.0(11)	1.1(11)	12.1(12)
O10	67.0(17)	79(2)	165(3)	26(2)	-11(2)	13.4(15)
C3	49.9(15)	40.0(14)	69.4(17)	6.9(13)	-0.6(14)	-2.1(12)
C7	51.5(14)	38.8(14)	43.3(13)	-4.4(10)	7.4(12)	3.3(12)
C1	45.3(15)	50.5(16)	52.4(14)	-18.1(12)	-4.1(12)	2.5(12)
C25	70(2)	48.9(17)	61.9(17)	16.9(14)	5.1(16)	8.7(16)
C2	60.1(18)	39.3(15)	79(2)	-14.8(14)	-1.2(16)	-4.3(13)
C13	55.1(17)	74(2)	43.0(13)	17.2(14)	-4.7(12)	11.4(16)
C12	63.2(18)	89(3)	36.7(13)	2.4(14)	-9.2(13)	4.1(18)
C11	59.7(17)	71(2)	39.4(13)	-10.9(13)	-6.4(12)	4.2(15)
C14	68(2)	83(2)	41.7(14)	16.4(15)	0.4(14)	5.1(18)
C15	54.0(16)	77(2)	44.8(14)	9.0(14)	4.6(13)	11.9(16)
C18	64.0(19)	53.4(18)	69.2(18)	5.1(15)	16.1(16)	-12.4(15)
C20	44.0(16)	68(2)	66.4(18)	4.6(15)	0.9(14)	17.9(14)
C26	56.2(19)	50.0(19)	131(3)	18(2)	-6(2)	7.5(16)
C19	86(2)	62(2)	49.8(15)	12.6(14)	-16.1(16)	-15.3(18)
O1	77(2)	78(2)	395(8)	101(4)	7(4)	13.5(19)
C24	54.6(19)	148(5)	70(2)	30(2)	9.6(18)	-15(2)
C28	132(4)	49(2)	108(3)	21(2)	14(3)	2(2)
C23	78(2)	114(4)	69(2)	36(2)	-17(2)	24(2)
C27	53.6(19)	79(3)	120(3)	17(2)	-6(2)	11.1(19)
C22	138(4)	136(4)	38.5(16)	-2(2)	-17(2)	-5(4)

Table S18. Bond lengths for andrastone B (**2**).

Atom	Atom	Length/Å	Atom	Atom	Length/Å
O3	C21	1.345(3)	C4	C19	1.529(4)
O3	C6	1.474(3)	C8	C17	1.562(4)
O8	C16	1.201(3)	C8	C7	1.560(3)
O2	C3	1.458(4)	C8	C20	1.558(4)
O2	C26	1.324(4)	C16	C17	1.559(4)
O4	C21	1.205(4)	C16	C15	1.516(4)
O6	C25	1.340(4)	C6	C7	1.519(4)
O6	C28	1.463(5)	C17	C25	1.545(4)
O5	C25	1.181(4)	C17	C13	1.591(4)
O9	C15	1.421(5)	C3	C2	1.515(5)
O7	C14	1.204(4)	C1	C2	1.520(5)
C9	C10	1.568(4)	C13	C12	1.538(5)
C9	C8	1.553(4)	C13	C14	1.529(5)
C9	C11	1.507(3)	C13	C23	1.544(5)
C5	C10	1.543(3)	C12	C11	1.319(5)
C5	C4	1.554(4)	C12	C22	1.520(4)
C5	C6	1.524(4)	C14	C15	1.522(4)
C10	C21	1.515(4)	C15	C24	1.501(5)
C10	C1	1.528(4)	C26	O1	1.177(5)
C4	C3	1.530(4)	C26	C27	1.481(6)
C4	C18	1.547(4)			

Table S19. Bond Angles for andrastone B (**2**).

Atom	Atom	Atom	Angle/°	Atom	Atom	Atom	Angle/°
C21	O3	C6	108.50(19)	C16	C17	C8	113.8(2)
C26	O2	C3	118.5(3)	C16	C17	C13	103.6(2)
C25	O6	C28	115.2(3)	C25	C17	C8	112.6(2)
C8	C9	C10	115.64(19)	C25	C17	C16	103.6(3)
C11	C9	C10	117.2(2)	C25	C17	C13	109.8(2)
C11	C9	C8	108.9(2)	O2	C3	C4	107.6(2)
C10	C5	C4	115.7(2)	O2	C3	C2	108.6(3)
C6	C5	C10	98.18(19)	C2	C3	C4	113.5(3)
C6	C5	C4	115.3(2)	C6	C7	C8	111.9(2)
C5	C10	C9	106.5(2)	C2	C1	C10	113.9(2)
C21	C10	C9	108.8(2)	O6	C25	C17	108.8(3)
C21	C10	C5	99.9(2)	O5	C25	O6	123.2(3)
C21	C10	C1	114.4(2)	O5	C25	C17	127.9(3)
C1	C10	C9	111.6(2)	C3	C2	C1	112.9(2)
C1	C10	C5	114.8(2)	C12	C13	C17	111.3(2)
C3	C4	C5	109.3(2)	C12	C13	C23	110.9(3)
C3	C4	C18	107.5(2)	C14	C13	C17	103.0(2)
C18	C4	C5	114.7(2)	C14	C13	C12	106.8(3)
C19	C4	C5	107.7(2)	C14	C13	C23	108.9(3)
C19	C4	C3	110.7(3)	C23	C13	C17	115.2(3)
C19	C4	C18	107.0(3)	C11	C12	C13	123.2(3)
C9	C8	C17	104.5(2)	C11	C12	C22	120.3(4)
C9	C8	C7	109.6(2)	C22	C12	C13	116.5(3)
C9	C8	C20	112.5(2)	C12	C11	C9	123.7(3)
C7	C8	C17	113.8(2)	O7	C14	C13	124.7(3)
C20	C8	C17	108.1(2)	O7	C14	C15	124.0(3)
C20	C8	C7	108.3(2)	C15	C14	C13	111.2(2)
O3	C21	C10	109.4(2)	O9	C15	C16	108.5(3)
O4	C21	O3	121.7(3)	O9	C15	C14	104.1(3)
O4	C21	C10	128.8(3)	O9	C15	C24	112.6(3)
O8	C16	C17	124.8(2)	C16	C15	C14	102.9(3)
O8	C16	C15	123.3(3)	C24	C15	C16	113.7(3)
C15	C16	C17	111.9(2)	C24	C15	C14	114.2(3)
O3	C6	C5	102.45(19)	O2	C26	C27	112.6(3)
O3	C6	C7	110.3(2)	O1	C26	O2	122.2(4)
C7	C6	C5	112.9(2)	O1	C26	C27	125.2(4)
C8	C17	C13	112.8(3)				

Table S20. Torsion angles for andrastone B (**2**).

A	B	C	D	Angle/°	A	B	C	D	Angle/°
O3	C6	C7	C8	53.4(3)	C6	C5	C10	C21	42.1(2)
O8	C16	C17	C8	47.3(4)	C6	C5	C10	C1	164.9(2)
O8	C16	C17	C25	-75.2(3)	C6	C5	C4	C3	-161.0(2)
O8	C16	C17	C13	170.2(3)	C6	C5	C4	C18	-40.2(3)
O8	C16	C15	O9	-77.0(4)	C6	C5	C4	C19	78.7(3)
O8	C16	C15	C14	173.2(3)	C17	C8	C7	C6	155.3(2)
O8	C16	C15	C24	49.1(5)	C17	C16	C15	O9	103.9(3)
O2	C3	C2	C1	62.9(3)	C17	C16	C15	C14	-6.0(4)
O7	C14	C15	O9	85.5(5)	C17	C16	C15	C24	-130.0(3)
O7	C14	C15	C16	-161.3(4)	C17	C13	C12	C11	0.1(4)
O7	C14	C15	C24	-37.6(6)	C17	C13	C12	C22	179.5(3)
C9	C10	C21	O3	83.8(3)	C17	C13	C14	O7	154.7(4)
C9	C10	C21	O4	-95.3(4)	C17	C13	C14	C15	-28.6(4)
C9	C10	C1	C2	-162.3(2)	C3	O2	C26	O1	-5.5(8)
C9	C8	C17	C16	54.9(3)	C3	O2	C26	C27	173.8(4)
C9	C8	C17	C25	172.4(2)	C7	C8	C17	C16	-64.7(3)
C9	C8	C17	C13	-62.8(3)	C7	C8	C17	C25	52.8(3)
C9	C8	C7	C6	38.6(3)	C7	C8	C17	C13	177.6(2)
C5	C10	C21	O3	-27.5(3)	C1	C10	C21	O3	-150.7(2)
C5	C10	C21	O4	153.4(3)	C1	C10	C21	O4	30.2(5)
C5	C10	C1	C2	-41.0(3)	C25	C17	C13	C12	158.4(3)
C5	C4	C3	O2	-65.5(3)	C25	C17	C13	C14	-87.5(3)
C5	C4	C3	C2	54.7(3)	C25	C17	C13	C23	31.0(4)
C5	C6	C7	C8	-60.5(3)	C13	C17	C25	O6	64.1(3)
C10	C9	C8	C17	-163.8(2)	C13	C17	C25	O5	-117.2(4)
C10	C9	C8	C7	-41.4(3)	C13	C12	C11	C9	1.5(5)
C10	C9	C8	C20	79.2(3)	C13	C14	C15	O9	-91.2(3)
C10	C9	C11	C12	-168.0(3)	C13	C14	C15	C16	21.9(4)
C10	C5	C4	C3	-47.2(3)	C13	C14	C15	C24	145.6(3)
C10	C5	C4	C18	73.6(3)	C12	C13	C14	O7	-88.0(5)
C10	C5	C4	C19	-167.5(3)	C12	C13	C14	C15	88.8(3)
C10	C5	C6	O3	-43.5(2)	C11	C9	C10	C5	-168.4(2)
C10	C5	C6	C7	75.1(2)	C11	C9	C10	C21	84.7(3)
C10	C1	C2	C3	48.4(4)	C11	C9	C10	C1	-42.4(3)
C4	C5	C10	C9	165.7(2)	C11	C9	C8	C17	61.9(3)
C4	C5	C10	C21	-81.2(3)	C11	C9	C8	C7	-175.8(2)
C4	C5	C10	C1	41.7(3)	C11	C9	C8	C20	-55.2(3)
C4	C5	C6	O3	80.1(2)	C14	C13	C12	C11	-111.6(4)
C4	C5	C6	C7	-161.3(2)	C14	C13	C12	C22	67.7(4)

C4	C3	C2	C1	-56.8(4)	C15	C16	C17	C8	-133.5(3)
C8	C9	C10	C5	61.1(3)	C15	C16	C17	C25	103.9(3)
C8	C9	C10	C21	-45.7(3)	C15	C16	C17	C13	-10.7(3)
C8	C9	C10	C1	-172.9(2)	C18	C4	C3	O2	169.4(2)
C8	C9	C11	C12	-34.4(4)	C18	C4	C3	C2	-70.3(3)
C8	C17	C25	O6	-169.4(2)	C20	C8	C17	C16	174.9(2)
C8	C17	C25	O5	9.3(5)	C20	C8	C17	C25	-67.6(3)
C8	C17	C13	C12	32.1(3)	C20	C8	C17	C13	57.2(3)
C8	C17	C13	C14	146.2(3)	C20	C8	C7	C6	-84.5(3)
C8	C17	C13	C23	-95.4(4)	C26	O2	C3	C4	-129.4(3)
C21	O3	C6	C5	28.6(3)	C26	O2	C3	C2	107.3(4)
C21	O3	C6	C7	-91.7(3)	C19	C4	C3	O2	52.9(3)
C21	C10	C1	C2	73.7(3)	C19	C4	C3	C2	173.2(3)
C16	C17	C25	O6	-46.0(3)	C28	O6	C25	O5	2.7(5)
C16	C17	C25	O5	132.7(4)	C28	O6	C25	C17	-178.5(3)
C16	C17	C13	C12	-91.5(3)	C23	C13	C12	C11	129.8(3)
C16	C17	C13	C14	22.7(3)	C23	C13	C12	C22	-50.8(4)
C16	C17	C13	C23	141.1(3)	C23	C13	C14	O7	31.9(6)
C6	O3	C21	O4	178.9(3)	C23	C13	C14	C15	-151.4(3)
C6	O3	C21	C10	-0.3(3)	C22	C12	C11	C9	-177.8(4)
C6	C5	C10	C9	-71.1(2)					

Table S_{21}. Hydrogen atom coordinates (Å×10^4) and isotropic displacement parameters (Å^2×10^3) for andrastone B (**2**).

Atom	*x*	*y*	*z*	U(eq)
H9	4291.59	3117.94	6949.8	136
H9A	6819.13	3391.09	6423.8	48
H5	6771.88	3863.86	5499.43	44
H6	8741.27	3096.01	4875.94	51
H10A	3106.15	4058.2	6280.6	155
H10B	4130.61	4717.43	6453.71	155
H3	7364.54	6519.41	5066.96	64
H7A	8924.41	1686.91	5484.97	53
H7B	7305.87	2061.99	5503.78	53
H1A	7025.13	5280.16	6498.84	59
H1B	8660.41	5470.21	6657.78	59
H2A	7618.43	6778.02	6075.25	71
H2B	9063.32	6340.09	5813.57	71
H11	8342.85	4155.77	7270.63	68
H18A	9994.51	5439.39	5104.26	93
H18B	9303.19	5780.63	4508.96	93
H18C	9765.71	4653.34	4599.95	93
H20A	10652.21	2412.51	6200.36	89
H20B	10148.98	1405.33	6497.47	89
H20C	10170.37	2410.6	6858.66	89
H19A	7265.84	4141.41	4312.01	99
H19B	6872.93	5283.82	4232.29	99
H19C	5908.03	4595.61	4633.26	99
H24A	3061.1	1172.4	7456.04	136
H24B	3837	449.76	7011.48	136
H24C	2904.18	1356.8	6781.15	136
H28A	6542.21	-1246.22	6132.64	144
H28B	5781.52	-1481.43	6731.53	144
H28C	7482.89	-1415.81	6696.63	144
H23A	8435.61	271.72	7444.54	130
H23B	8449.95	914.06	8020.65	130
H23C	9591.88	1129.71	7526.38	130
H27A	3016.28	5630.37	5145.14	127
H27B	2623.78	6736.04	5330.14	127
H27C	3145.55	5958.14	5802.42	127
H22A	7273.49	2722.18	8362.3	156
H22B	8214.41	3680.09	8225.8	156
H22C	8965.94	2625.43	8295.83	156
